# Guideline for software life cycle in health informatics

**DOI:** 10.1016/j.isci.2022.105534

**Published:** 2022-11-09

**Authors:** Anne-Christin Hauschild, Roman Martin, Sabrina Celine Holst, Joachim Wienbeck, Dominik Heider

**Affiliations:** 1 Department of Data Science in Biomedicine, Faculty of Mathematics & Computer Science, Philipps University of Marburg, 35043 Marburg, Germany; 2 Department of Medical Informatics, University Medical Center Göttingen, Georg-August-Universität, 37099 Göttingen, Germany

**Keywords:** Health informatics, Bioinformatics, Software engineering

## Abstract

The long-lasting trend of medical informatics is to adapt novel technologies in the medical context. In particular, incorporating artificial intelligence to support clinical decision-making can significantly improve monitoring, diagnostics, and prognostics for the patient’s and medic’s sake. However, obstacles hinder a timely technology transfer from research to the clinic. Due to the pressure for novelty in the research context, projects rarely implement quality standards.

Here, we propose a guideline for academic software life cycle processes tailored to the needs and capabilities of research organizations. While the complete implementation of a software life cycle according to commercial standards is not feasible in scientific work, we propose a subset of elements that we are convinced will provide a significant benefit while keeping the effort within a feasible range.

Ultimately, the emerging quality checks for academic software development can pave the way for an accelerated deployment of academic advances in clinical practice.

## Graphical abstract

**Figure:**
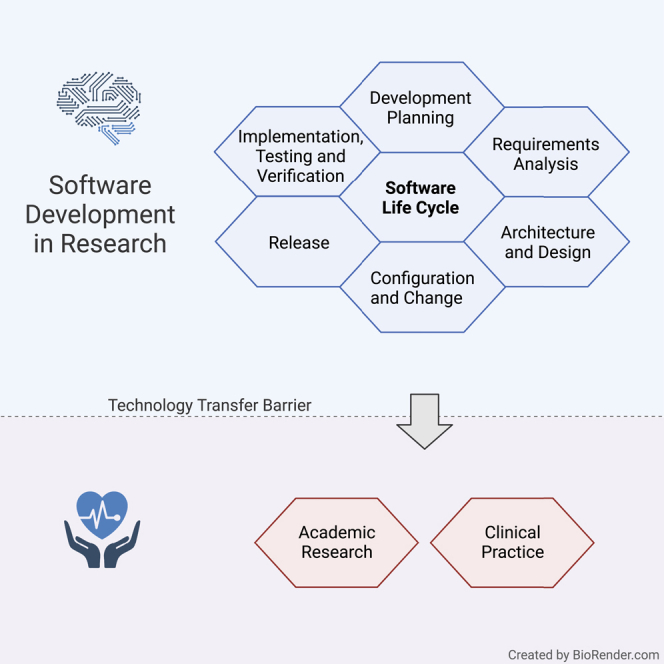

## Teaser

Health informatics; Bioinformatics; Software engineering; standardization

## Introduction

Today, medical informatics is an integral part of health care systems that ensure the smooth operation of processes in medical care. Moreover, standard procedures ensure the transfer of knowledge from medical research to clinical practice, for instance, via regularly updated guidelines and regulations. In contrast, newly developed software innovations such as artificial intelligence-based systems that could support clinical decisions and have already evolved to be state-of-the-art in medical informatics research rarely transfer to application in practice.

Modern methods such as artificial intelligence (AI) and machine learning increasingly unroll their potential in medical healthcare to help patients and clinicians.^1^ Clinical decision support systems (CDSS) can effectively increase diagnostics, patient safety, and cost containment.^2^ Easy accessible AI-based applications to patients can improve diagnosis and treatment, e.g., by precisely detecting symptoms,^3^ evaluating biomarkers,^4^ or detecting pathogenic resistance or subtypes.^5^^,^^6^ Furthermore, upcoming concepts such as Federated Learning^7^^,^^8^ and Swarm Learning^9^ allow the cross-clinical creation of data-driven models without bursting patient’s privacy,^10^^,^^11^ opening the gate for more powerful data-driven development.

To protect patients from any risk of injury, disability, or other harmful interventions, the described developments, so-called medical device software (MDSW), which is intended to provide specific medical purposes, such as diagnosis, monitoring, prognosis, or treatment, underlies strict regulations. For instance, the European Medical Devices Regulation (MDR),^12^ the *In Vitro* Diagnostic Medical Devices Regulation (IVDR),^13^ or the International Medical Device Regulators Forum (IMDRF).^14^

MDSW can be an integral part of a medical product or standalone software as an independent medical device.^15^ Moreover, it is irrelevant for qualification as MDSW whether the software runs in the Cloud, a platform, or a server and whether healthcare professionals or laypersons use it. Exceptions are software tools used for documentation or that solely control medical device hardware or with no medical purpose.^13^

An integral part of all regulations for MDSW is the development according to the software life cycle process as defined by IEC 62304,^16^ a harmonized standard that regulates required documentation and processes, such as software development planning, requirement analysis, architectural design, testing, verification and maintenance.^12^^,^^17^

These regulations focus on minimizing patient risk, for example, harmful follow-up analysis, a wrong or missing treatment where needed, as a result of software failures, incorrect predictions, or other malfunctions. Several challenges arise to eliminate these risks, allowing for a smooth technology transfer and reproducibility.

### Challenges of scientific software development for health

The primary goal of scientists remains to do science rather than the developing software. However, many scientists aim to make their findings and methodologies available to a broader audience and to be used for the greater good. Thus, many data science and AI methodologies, as well as corresponding implementations and software packages, exist that would, in theory, allow the development of efficacious AI-driven medical decision support systems. However, scientists of different backgrounds tend to have very different knowledge of software engineering practices, often acquired through self-study.^18^ Moreover, academic groups often consist of small teams that undergo frequent change or researchers that work on a one-person-one-project basis.^19^^,^^20^ Thus, a lack of attention to relevant software development processes and engineering practices defined by the software life cycle negatively affects the usefulness of developed packages, particularly for developing software as a medical device.^21^

Pinning down the most critical requirements along with an accurate description and documentation of such is a significant challenge for all software projects independent of if it is conducted in research or industry.^18^^,^^22^ It necessitates a detailed analysis of the non-functional requirements as determined, for instance, by regulatory entities such as security, privacy, or infrastructural limitations, as well as functional requirements like user-friendly interfaces and specific results. This is very time-consuming and relies on a close interaction of developers, stakeholders, and potential users, which is often difficult to achieve under academic circumstances.^20^^,^^22^ Moreover, researchers are enticed by academic hiring procedures, and funders to focus on “novelty” rather than software quality and practical usefulness.^19^^,^^20^ Thus, implementations often fail to fulfill requirements, ensuring long-term sustainability such as documentation, usability, appropriate performance for practical application, user-friendliness optimally supporting potential users, and minimizing risks.^19^^,^^23^

The most critical aspects of ensuring sustainability in academic software are reproducibility, reusability, and traceability.^21^^,^^24^ However, it has been shown that not only public accessibility but also documentation and portability are essential to ensure reproducibility and underpin trust in the scientific record of scientific software enabling the re-use of research and code. Moreover, the prototype-centered development procedures often lack quality checks, such as systematic testing, that would ensure reusability.^21^ Recently, scientific journals such as GigaScience or Biostatistics have promoted reproducibility and reusability by mandating FAIR principles’ (i.e., Findability, Accessibility, Interoperability, and Reusability). FAIR establishes a guideline for scientific data management and documentation.^19^^,^^25^ Implementing the FAIR principles in academic software development as an excellent scientific practice lowers the barriers to a successful industrial transition.

Additionally, for long-term software maintenance, well-structured development planning and processes can ensure the traceability of modifications via change management and version control. These aspects ultimately determine scientific rigor, transparency, and reproducibility.^19^

### Software life cycle

The software life cycle (SLC) process guarantees high-quality planning, development, and maintenance of a SaMD. It covers the planning and specification, development, maintenance, and configuration. The IEC 62304 defines the software life cycle (SLC) as a conceptual structure, including its lifetime from the requirements’ definition until the release. It describes processes, tasks, and activities involved in developing a software product and their order and interdependencies. Furthermore, it defines milestones verifying the completeness of the results to be delivered.^16^

However, a complete software life cycle (SLC) described in these standards is not feasible for most research projects.^26^ Academic research is often subject to tight schedules and focuses on proof of concept development, neglecting formal documentation or procedures. Here we provide recommendations for an SLC in academia, lowering the boundary for many research organizations to implement an SLC and fostering the transfer of technology to industrial development.^13^^,^^26^

### Our goal: Academia-tailored software life cycle

Until now, there is little guidance on supporting a structured software development culture for academic institutions according to standard software life cycle processes. In this article, we present a synopsis of all requirements in official standards that are relevant to academia. We adjusted these toward the specific demands of software development in research and established a limited software life cycle process for research organizations, which has the potential to greatly facilitate and speed up such technology transfer and reproducibility in a controlled and predictable way. Being aware that a complete software life cycle is not feasible for most academic settings, we proposed a subset of elements that we are convinced will provide a significant benefit without creating an excessive organizational burden for researchers and developers and keep activities in a manageable range.

Our proposal is centered on procedures for software development planning, software requirement analysis, software architectural design, software unit implementation, integration, testing, verification, and configuration management. Depending on the specific needs, the elements of a software life cycle process that work best for an organization may differ from what we propose. The fact, however, that a life cycle process is set up at all and that the elements are deliberately chosen is probably a key factor for facilitating technology transfer. However, any medical software development for clinical use must strictly follow the regulations relevant to the specific country or region. Thus, our guideline can only provide a starting point intended to be adapted to institute- or project-specific requirements, considering only relevant aspects. Nevertheless, the ideas presented here are not meant to provide a shortcut for medical software. An overview of our suggested SLC activities in comparison with the regulatory is provided in Table S1.

## Software life cycle for medical software research guideline

**Figure 1:**
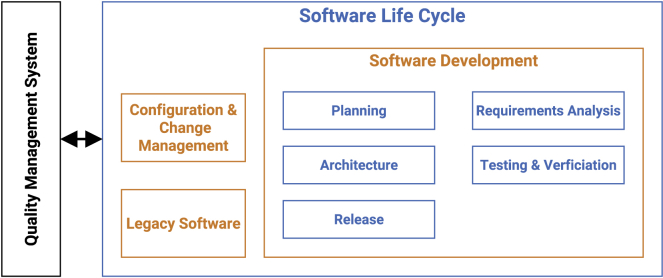
Main components of software life cycle The main focus of the Software Life Cycle in compliance with the Quality Management System lies in the Software Development but integrates Configuration and Change Management as well as Legacy Software.

Our guideline covers multiple processes such as Development Planning, Requirement Analysis, Software Architecture, Software Design, Implementation, Software, and Integration Testing, Verification, and Release. In the following, we present the most vital points for the software life cycle as depicted in Figure 1.

### Software development planning

Defining an accurate Software Development Plan is the first key component and must be updated regularly during the project, referenced, and defined in the entire software life cycle model. It defines norms, methods, used processes, deliverable results, traceability between requirements, software testing, implemented risk control measures, configuration and change management, and verification of configuration elements, including software of unknown provenance (SOUP). SOUP is a commonly used basic software library or packages that have not been developed for medical purposes.

Two documents should be provided for the academic tailored implementation: the process description and the development plan. Each document produced during the software development has to contain a title, purpose, and responsible person.^16^ In the case of multiple involved developers, the role and responsibility assignments should be noted down in the single process description.

As a general handbook, the process description is provided by the definition of standard operating procedure (SOP) solutions for repetitive application problems.^27^ The description contains each activity within the processes when it will be completed, by whom, how, and with which in- and output. The developed standard process description can be applied to multiple projects and has to be implemented by the developers.^12^

Since the life cycle model must be completely defined or referenced by the development plan, a software engineering model must be selected to provide a general structure for the development phase, such as the V-model. As a common approach in medical device development, the V-model is successfully used to achieve regulatory conformance.^28^ Generally, the selected model should match the project’s characteristics and thus can host agile practices or elements of SCRUM to support life cycle conform development.^29^^,^^30^

The development plan process describes the general documentation required for a product or project. The development plan defines concrete milestones to corresponding deadlines, assigns concrete staff to the pre-defined roles, and refines measures or tools adjusted to the concrete project. It is challenging to meet the regulatory requirement of defining tools, testing, and configuration management in advance, particularly in a volatile academic setting. Due to external factors and unforeseeable changes, the development plan must be updated over the project while the pre-defined process descriptions remain. Additionally, the process should recommend defining a coding guideline or convention, including code style, nomenclature, and naming in the development plan, to increase software quality. For example, pre-defined rules in git hooks or Continuous Integration (CI) combined with linting tools can enforce coding compliance. The development process gets more transparent and well-structured through the provision of those two documents.

### Software requirements analysis

A software requirement is a detailed statement about a property that a software product, system, or process should fulfill and is defined in the Software requirements analysis. Software requirements should cover functional requirements such as inputs, outputs, functions, processes, interface reactions, and thresholds, as well as non-functional requirements such as physical characteristics, computing environments, performance, cybersecurity, privacy, maintenance, installation, network, and so forth. Precisely defined requirements are a vital element for the success of a project since studies concluded that half of all software errors are derived from mistakes in the requirement phase.^31^^,^^32^ High-level requirements should be defined within the specifications, including the desired properties. These specifications describe mainly the project’s total goal under defined restrictions. For practical consideration, it is beneficial to begin with general natural language requirements and refine them into graphical notations, such as Unified Model Language (UML) diagrams.^33^^,^^34^

However, the original requirement within the specification must always be bidirectionally linked to allow traceability. It should be possible to describe and follow the life of requirements in all directions. This means that traceability implies the comprehension of a design, starting with the source of a requirement, its implementation, testing, and maintenance. Moreover, it facilitates high software quality, a critical concern for medical devices. Therefore, the derivation and documentation of the requirements should be updated and verified during the project.^16^ Finally, a critical aspect is the definition of requirements for maintenance, which is often neglected in academia since the focus is on publishing new technologies in contrast to maintaining existing software.

Nevertheless, maintenance must be considered at the beginning of the software life cycle to ensure that post-delivery support is possible.^35^ Therefore, a maintenance plan in academia does not have to be complete but must define all factors influencing either the development or architecture. Table 1 can be used to evaluate the completeness of a software requirement analysis. Further, an implementation example is provided in the supplemental information.

**Table 1:** The product quality properties by ISO 25010 to evaluate if the software requirements are complete.^36^

Usability	Functional Suitability	Security	Maintainability
- Appropriateness Recognizability - Learnability - Operability - User Error Protection - Accessibility	- Functional Completeness - Functional Correctness - Functional Appropriateness	- Confidentiality - Integrity - Non-Repudiation - Accountability - Authenticity	- Modularity - Reusability - Analyzability - Modifiability - Testability
Performance Efficiency	Reliability	Portability	Compatibility
- Time Behavior - Resource Utilization - Capacity	- Maturity - Availability - Fault Tolerance - Recoverability	- Adaptability - Installability - Replaceability	- Co-Existence - Interoperability

### Software architecture and software design

The regulatory authorities demand the definition of essential structural software components, identification of their primary responsibilities, visible features, and their interrelations.^16^ The architecture as an overarching structure conceptually defines data storage, interfaces, and logical servers. At the same time, modularization is described within the detailed software design, specifying how the single elements of the architecture and the requirements are explicitly implemented.

Software architecture consists of the system’s structure in combination with architecture characteristics the system must support (e.g., availability, scalability, and security), architecture decisions (formulation of rules and constraints), and design principles. An architecture categorizes into monolithic and distributed architecture types consisting of single packages or separatable sub-systems.^37^ It is recommended to specify an appropriate architecture prior to implementation. However, the choice is not regulated.^16^ These architectural design decisions will guide the developers throughout the development process.

The system has to be divided for the software design until it is represented through software units. These are sets of procedures or functions encapsulated in a package or class that cannot be further divided. Each software unit and interface needs a verified detailed design to ensure correct implementation.^16^ The design principles cover every option or state of all system components in detail, such as a preferred method or protocol.^38^ In order to have well testable and maintainable code, it is recommended to have software with low coupling (dependencies between the sub-systems) and high cohesion (internal dependencies).^39^ These associations can be well described using widely accepted notation standards such as UML, including class and activity diagrams to document the architectural decisions, which is highly recommendable to facilitate understanding the architecture.^40^

In academia, two documents should be provided: the software architecture description and the detailed design. It is unlikely to narrow down the whole codebase in a detailed design. However, it must contain the utmost vital components, such as elements of design patterns, classes with crucial functionality, or specific interfaces. For example, a strict logical dissociation between the internal logic and the interface, e.g., for user interaction, is critical. Design patterns such as the model view controller (MVC) are favorable.^41^

### Implementation, testing, and verification

Generally, each software unit must be implemented, tested, and verified. The IEEE defines implementation as translating a design into hardware or software components, or both.^42^ In particular, the detailed design has to be translated into source code. Following a specific coding style and documentation standards is advisable during the implementation. Subsequently, every software unit has to be tested and verified separately, ensuring it works as specified in the detailed design and complies with the coding style. After that, it has to be integrated, verified, and tested dependently and independently in the following integration tests. These evaluate the software unit’s functionality combined with other components into an overall system.

The software is usually tested on different abstraction levels within the software’s life cycle, differentiating between unit, integration, regression, and system testing.

While isolated unit tests verify the functionality of a separately testable software element, integration tests verify the interaction between the software units, as described by the software architecture. Along with different test strategies, such as top-down or bottom-up, integration tests must be conducted during several stages of the development process and are tailored to each integration level.^43^

Integration and system tests can be combined with routine activities but must cover all software requirements. Especially, software components affecting safety require extensive tests. Appropriate evaluations of the testing procedure, verification, and integration strategy concerning the previously determined requirements are necessary. Tests and results must be recorded with acceptance criteria, providing repeatability and traceability between requirements and their verifications.

Tests can be performed either as white-box testing,^44^ including the knowledge of the underlying architecture or as black-box testing,^45^ which does not take into account the internal structure. Besides automated tests, non-automated tests should be conducted between program coding and the beginning of computer-based testing. Moreover, the three fundamental human testing methods are inspections, walkthroughs, and usability testing.^46^ As demonstrated in our example in the supplemental information, SCRUM supports software integration and system testing since, after each sprint, an increment of potentially shippable functionality consisting of tested, well-written and executable code is required (Figure S2). Consequently, verification and testing are automatically included in the process of SCRUM. Project-specific regular, complete tests which are documented and traceable to requirements, software architecture, and detailed design are critical for software development within the law. A test is successful if it passes the acceptance criteria, defined through the requirements specification, the interface design within the detailed design, and the coding guideline. Ultimately, the verification evaluates whether all specified requirements are fulfilled by validating objective proof.^16^

To ensure adequate software verification, it has to be well planned and integrated into several stages of the SLC: requirements analysis, software architecture, software design, and software units, as well as their integration, changes, and problem resolutions, have to be verified. The management of verification documents can also be partially organized automatically through CI or as such with the Jira API or the Gitlab CI/CD. Table 2 lists the suggested aspects to verify the different stages of the software development process. In particular, problem resolutions and other changes have to be re-verified and documented. Overall, verification is an activity of high importance throughout the whole development process. To verify more mature artifacts, one must verify their foundation as well. This hierarchy should always be kept in mind, as the supplemental information example demonstrates.

**Table 2:** Verification at all software development process stages

System requirements	Software requirements
- Must be derived from the stakeholder’s requirements - May not contradict each other - Must be consistent, unambiguous, clearly identifiable	- Implement the system requirements - May not contradict each other - Must be consistent, unambiguous, clearly identifiable - Must be traceable to the system requirements or other sources - Testing criteria must be driveable
Software Architecture	Detailed Design
- All System and software requirements are implemented - Must support the interfaces as well as SOUP items	- Implements do not contradict the software architecture.
Software Units	Software Integration
- Test case for each requirement must be passed - Code may not contradict the interface design, the detailed design, or the coding guideline - Verification of all requirements, architecture, and detailed design must be documented	- Software unit integration is realized according to an integration plan derived from the software architecture - Software system tests verify the software’s functionality

### Software release

In contrast to industry, academic software is released to other researchers via public repositories and journal publications. Before software release, testing and verification need to be completed and evaluated. That includes, first, all known residual anomalies that have to be documented and evaluated. Second, documentation of the release procedure and the software development environment has to be recorded with the released software version. Third, all activities and tasks of the software development plan must be completed and documented. Fourth, the medical device software, all configuration elements, and the documentation must be filled for the whole lifetime of the medical device software, defined by the development team as long as the relevant regulatory requirements demand it. Fifth, procedures to ensure a reliable delivery without damaging or unauthorized adjustments have to be defined.^16^

After the software is released, all changes and updates are implemented within the software maintenance process, following the same steps as the software development process. The post-delivery maintenance decisions that must be made are included in the software development planning.

Suppose the development process is well defined and followed, and the previous sections of this guideline are considered. In that case, the complete verification and the required documents are delivered by default. Well-implemented traceability is essential to ensure the development process can be archived transparently. Regarding SCRUM, one could include the required documentation within the definition of done to ensure everything is documented since the development takes place in a regulatory context.

### Legacy software

According to IEC 62304, legacy software is defined as software that was not developed to be used within software as a medical device, such as general software packages and libraries. It, therefore, lacks sufficient verification that it was developed in compliance with the current version of the norm.

Thus, it is sufficient to prove it conforms to the norm, and shortcomings to the norm’s requirements need assessment.^16^ Hence risks of using the legacy software, as well as the risks of missing documentation, need to be identified and mitigated, if possible, as defined by the risk management process in IEC 62366-1.^47^

In academia, it is essential to choose legacy software and document its usage carefully. Ideally, the used legal software has to fulfill the requirements of the IEC concerning risks as well, but the scope of action only demands closing gaps if it reduces the risk of usage.

### Configuration and change management

Configuration and change management is crucial in ensuring usability, reproducibility, reusability, and traceability of software in the industry and academia. In academic research, automated change management systems, such as GitHub or GitLab, exist for software code and data and are regularly used.^25^ However, implementing adequate configuration and change management documentation for the entire software life cycle, as required by the IEC 62304, is particularly challenging in academia, where the pressure to publish urges researchers to focus on novelty rather than maintenance.

In order to mitigate the ongoing replication crisis,^48^^,^^49^ academic research institutions and projects should establish technical and administrative procedures to identify and define configuration items and SOUP and their documentation within a system. This should include the documentation of problem reports, change requests, changes, and releases necessary to restore an item, determine its components and provide the history of its changes. In particular, configuration change requests need to be documented, approved, and verified in projects with multiple developers and stakeholders.^16^ Thus, it is advised to assign a representative person, ideally permanent technical academic staff, in charge of the change management to support the correct implementation of necessary processes for groups and projects in advance.^23^ In academia, ticket systems such as those provided by most repositories can be easily used as version control systems for all code and documents to facilitate tracking changes. Moreover, tools such as Jira for project management and Confluence for project documentation are advisable to ensure traceability and good configuration management.

These tools can be utilized to establish a change management strategy or process that should include steps like:

1. Create a problem report (including criticality)
2. Problem Analysis, including software risk
3. Create a change request if required
4. Implementation and verification of the change

## Discussion

The proceeding trend toward new technologies in the medical context,^1^ such as establishing AI- or machine learning-derived software, paves the way to significantly improve monitoring, diagnostics, and prognostics for the patient’s and medic’s sakes. The subjacent development, realized mainly by specialized research organizations and institutes, is time-consuming. Additionally, implementing these software achievements in the medical markets requires enormous efforts to cover all requirements of international standards.^19^^,^^20^^,^^21^ This article reveals several knowledge transfer challenges from academia to industrial standards, focusing on the software life cycle in a biomedical context. Challenges mainly face the software development and engineering processes regarding reproducibility, reusability, and traceability.

Here, to establish an academia-tailored software life cycle, we propose a comprehensive guideline for research facilities derived from the requirements of IEC 62304.^16^ Complementary to our quality management guideline,^50^ we propose to address these challenges by following our guideline, which lowers the barriers to a potential technology transfer toward the medical industry. Furthermore, in the supplemental information, we provide a comprehensive checklist for a successful Software Life Cycle and demonstrate the feasibility of our guideline with our implementation example.

Since realizing the regulatory requirements is mostly not feasible in an academic context, we focus on the most vital aspects of the SLC, covering software planning, development, architecture, maintenance, and legacy software. The implementation of our guidelines will not only improve the quality of software and avoid engineering errors but also mitigate potential risks that might arise from the introduction of AI in healthcare. An integration of SLC as standard procedure in academic programming could increase the overall quality of processing pipelines and thus the quality of data, evaluation strategies are well planned leading to a closer look at potential risks of false positives and false negatives. Ultimately, this will support a smooth transfer with potential manufacturers by delivering all demanded documents of a certain quality in the software. Although some research organizations realize industrial standards such as quality management,^51^ we encourage scientists to further introduce software life cycles as a usual practice for software in research institutes. We envision that such SLC in addition to the FAIR principles for scientific data management and documentation could become the standard for scientific health software publishing. Finally, this may pave the way for a smoother transition from research toward clinical practice.
