# Supplementary material for: Guideline for software life cycle in health informatics
Source: iScience. 2022 Nov 9;25(12):105534. doi: 10.1016/j.isci.2022.105534 (PMC9685384; doi:10.1016/j.isci.2022.105534)
Supplement: Document S1. Figures S1–S8 and Tables S1–S7 [file mmc1.pdf]

iScience, Volume *25*

Supplemental information

Guideline for software life cycle in health informatics

Anne-Christin Hauschild, Roman Martin, Sabrina Celine Holst, Joachim Wienbeck, and Dominik Heider

Software Life Cycle Process Short Checklist **2**
Checklist 2
Before the development starts 2
Before the implementation starts 3
During the implementation 3
After the implementation 3
Functional Implementation Tips 4
Software Life Cycle Activities Comparison **5**
An implementation example of a Software Life Cycle **6**
Software Development Planning 8
Software Requirement Analysis 14
Software Architecture and Software Design 18
Implementation, Testing and Verification 26
Appendix **31**
A.1 Additional Material 31
B Outputs of the Project Development Plan 37
B.1 Requirement Specification 37
B.2 Software Architecture Description 41
B.3 Software Architecture Description 42
B.4 Mockup 47
B.5 Coding Guideline 48
B.6 Sprint Notes 50
B.7 Verification Documents 51
B.8 Change Requests 64

Software Life Cycle Process Short Checklist

The following guide shows the essential key steps to developing a software life cycle (SLC). It provides a low-barrier entrance point for academic researchers to follow a tailored SLC process. The guide is divided into a checklist and practical implementation tips. The list explains adequate consideration and timing of the regulatory requirements, whereas the implementation tips give concrete suggestions on how those requirements can be realized within an academic project.

Checklist

Follow this checklist to ensure a life cycle compliant software development sufficing the IEC 62304. Consider that in case of an intended formal admission and release of the software, strict compliance with the regulatory requirements of the notified bodies must be ensured as well as the possible need for a clinical evaluation.

1. State the intended purpose of your product (critical for medical device qualification).
2. Describe the process and classification (e.g., IEC 62304, MDR, or IVDR) used for software safety class determination.
3. Describe the software development process and how it is implemented within the project concerning a specific safety class. For class C of IEC 62304, this would include steps before the development starts, before the implementation starts, steps during and at the end of the implementation.

Before the development starts

1. Define a software development plan, reference a life cycle model, and include the development activities, their results, traceability, and the tools used.

The referenced activities are at least: software development planning, software requirement analysis, architectural software design, software unit implementation, integration, and testing, as well as verification, release, configuration management, and problem-solving processes.

1. Determine your stakeholders, the appropriate requirement elicitation technique, and find a structure for your requirement specification.
2. Describe the software architecture and design process, define a certain description structure or UML, how detailed you want to document and if you must consider SOUP or legacy software.
3. Define how you will verify all your results, including the appropriate tests and their documentation. The tests must cover all software requirements.
4. Define the conditions that must be met for a software release, including completing and documenting all activities within the development plan. Define how long your software and its documentation must be filed and how reliable delivery can be ensured.

5. Define a configuration management process. This should determine what information needs to be documented in the software configuration and change documentation to ensure traceability within the project.
6. Define the software problem-solving process, which should determine how internal and external problems are handled. Moreover, it should be defined what information is collected and how corresponding changes are documented and communicated within the project.

Before the implementation starts

1. Conduct a requirements elicitation.
2. Consider software maintenance requirements at the beginning of the project.
3. Define high-level requirements first.
4. Define a coding guideline.

During the implementation

1. Refine the high-level requirements.
2. Document all your architectural and design decisions. Each software unit and interface needs a detailed design to ensure correct implementation.
3. Always ensure traceability between your implementation, its verification, the requirements specification, and architecture and design documents.
4. Document the software testing and verification process and test all software units specified within the software architecture.

After the implementation

1. Document the software release, all remaining anomalies, and all third-party libraries used to ensure future observation.
2. File the software, the configuration elements, and the documentation.

Functional Implementation Tips

A law-compliant software development plan defines done (DoD), system and integration testing, and a problem-solving process. Additionally, two tables describe how verification can be ensured throughout the project. It is defined how to guarantee configuration management and traceability using Confluence and Jira for documentation and project management.

- Possible software development plan that could be adjusted.
Within this project, a requirement is fully implemented, sufficing the DoD if:

- The code is coding guideline compliant.
- The code is consistent, unambiguous, and identifiable.
- A code review was conducted.
- All acceptance criteria are fulfilled.
- A build has been made and deployed in a testing environment.
- All necessary unit, integration, and system tests are passed and documented.
- Test methods are appropriate, passed, and cover all afflicted requirements.
- Traceability to the requirements specification, epics, architecture, tests, and the detailed design document is implemented.
- The implementation does not contradict the architecture description or detailed design document.
- Sprint notes do exist.
- The documentation is complete.
- Problems found during unit integration and system testing are imported into the problem-solving process.

- Create a project space in Confluence, including:

- A shortcut to project management in Jira
- A development plan
- A requirement specification page
- A coding guideline page
- A software architecture description page
- A detailed design document page
- A sprint notes page
- A verification documents page
- A problem report page
- A change requests page

- Manage your project in Jira and link the issues with the documentation in Confluence
- To verify all artifacts within the development process (Table S2)
- The verification documents must prove or include (Table S3)
- Problem-solving process definition (Figure A2)
- System and integration testing definition (Figure A3)

Software Life Cycle Activities Comparison

Table S1. Overview of Software Life Cycle activities required from IEC 62304 compared with the concentrated activities suggested for the Academia within this manuscript.

**Activity**	**IEC 62304**	**Academia**
Software Development Plan	5.1	**2.1** Software Development Planning
Software Requirement Analysis	5.2	**2.2** Software Requirement Analysis
Software Architecture	5.3	**2.3** Software Architecture and Software Design
Software Detailed Design	5.4	
Software Unit Implementation and Verification	5.5	**2.4** Implementation, Testing and Verification
Software Integration and Integration Testing	5.6	
Software System Testing	5.7	
Software Release	5.8	**2.5** Software Release
Software Legacy Software	3.29	**2.6** Legacy Software
Software Maintenance	6	**2.7** Configuration and Change Management
Software Configuration Management	8	
Software Problem Resolution	9	
Software Risk Management	7	**-**

An implementation example of a Software Life Cycle

In the following, we will describe the development of a mobile application for type 2 diabetes diagnosis, using the SLC guideline from section three. It will be developed in an academic scope and can be considered a proof of concept, verifying the adaptability of the developed guideline. Moreover, our app development should contribute examples of the principles and concepts mentioned within the guideline, making them more concrete and easier to use. The implementation part will not be used only to apply the guideline but to revise and improve it. Consequently, the app development goal will not be publishing the app within a store, which would not be allowed for medical device software without a CE Mark. Its aim will be the guideline - and therefore IEC 62304 - compliant development of MDSW within an academic scope.

The theoretical background of this thesis already stresses the criticality of early type 2 diabetes diagnosis. Early diagnosis can prevent severe damage to organs and the body, which, in turn, could prevent high healthcare costs for patients and the medical system. Moreover, mobile support through apps for diabetes patients is already quite familiar. A diagnosis-support app might increase both the awareness of type 2 diabetes and the trust of potential users since data will be stored locally. Online tests for type 2 diabetes risk estimation, like the FINDRISK by the Deutsche Diabetes Stiftung[1] or the 60-second type 2 diabetes risk test by the American Diabetes Association, do already exist[2]. However, studies show some advantages of apps compared to websites. Apps can only use some features of mobile devices since they can access the phone's hardware, such as the speakers or the camera. Apps are well-integrated into the mobile device environment, improving usability and performance since they are fitted to the manufacturer's hardware using its designated technologies. The possibility of publishing the app on the manufacturer's marketplaces, like the Google Play Store or the Apple App Store, increases the app's discoverability because new user groups can be accessed[3]. Furthermore, apps can be accessed offline, and they can be opened through one tap instead of browsing search engines. Moreover, they are modern and state-of-the-art[4].

Since the declared purpose by a software manufacturer determines its qualification as MDSW, we will state the app's intended purpose as follows: The app's purpose is to support doctors and patients with the diagnosis of type 2 diabetes. It calculates a risk score that considers the known factors influencing the risk of developing type 2 diabetes. However, for a final valid diagnosis, blood tests are mandatory. Moreover,

---

1 DDS. 2022. *Deutsche Diabetes Stiftung. GesundheitsCheck DIABETES (FINDRISK)*. Deutsche Diabetes Stiftung. https://www.diabetesstiftung.de/gesundheitscheck-diabetes-findrisk.

2 ADA. 2022. *American Diabetes Association. Our 60-Second Type 2 Diabetes Risk Test.* American Diabetes Association. https://www.diabetes.org/risk-test.

3 Erlenwein, Thomas, Jürgen Karla, and Dennis Maus. 2020. 'Mobile Anwendungen und die Entwicklung der App Economy'. In *Handbuch Digitale Wirtschaft*, edited by Tobias Kollmann, 81–103. Wiesbaden: Springer Fachmedien.

4 Arroyo-Vázquez, Natalia, and José Antonio Merlo Vega. 2017. 'Comparing the usage data of an app and a mobile website for an academic library'.

one can use the collected information to initiate necessary lifestyle changes, such as increased physical activity or a healthy diet. Consequently, the software is MDSW, but more precisely, it is SaMD since it is not part of a hardware medical device. Therefore, one must suffice the life cycle-compliant software development required by the MDR.

For that reason, the software development takes place within a legal framework mainly defined by the MDR and the IEC 62304. The European market is focused, although the IEC 62304 is a recognized consensus standard by the FDA. These laws are constraints for software development and must be considered within the requirements specification. Since the guideline's development was based on the activities required by those laws, its appliance ensures a development within the law. Nevertheless, the guideline does not replace existing laws, which must be complied with to pass an audit and to obtain the CE Mark that allows for the official marketing of the product within the EU.

We used a classification scheme to determine the appropriate safety class for the app. A hazardous situation could occur if the app calculates an incorrect score. If the calculated risk is too low, no further examinations will follow. The disease could stay undiagnosed, causing potential long-term damage to the heart, blood vessels, kidneys, eyes, and nerves. If the calculated risk is too high, the patient would be further examined based on the risk score, although those examinations would be unnecessary. Blood tests, or other examinations in general, are an unnecessary risk to patients.

Nevertheless, a doctor will supervise the patient, who will evaluate the delivered risk score before performing any examination and potentially detecting risk factors or symptoms themselves. Control through a human being, who studied medicine for several years, is an external risk control measure. Consequently, the software does not lead to an unacceptable risk after considering the external risk control measures and can be classified as software safety class A. However, the guideline states that the requirements of the IEC 62304 for software class A do not suffice the demanded life cycle compliant development by the MDR. It is uncertain in which context the app will be reused; it could be enhanced within the working group or the FeatureCloud project. The data collected through the questionnaire could be used to train a machine learning algorithm, allowing more severe diagnoses within clinical practice without the control of a medic. Consequently, the software will be treated as safety class C during the development, as suggested by the guideline, to ensure regulatory compliance and to enable the reuse of the software within another context without the need to reproduce missing documentation.

Software Development Planning

Software development planning is a very complex activity within the SLC. Initially, we used a turtle diagram to identify the relevant process elements. Since this is an optional supporting tool for process definition, see Figure A1.

Later, we conceptualized a general process description as an SOP which could potentially be reused within other projects. It is inspired by the AV-model, depicted in Figure S1, and published by McHugh et al [5]. It combines the V-model within Figure 5 with Scrum. The AV model was developed and implemented by the high-tech product development and design engineering consultancy firm BlueBridge Technologies [5].

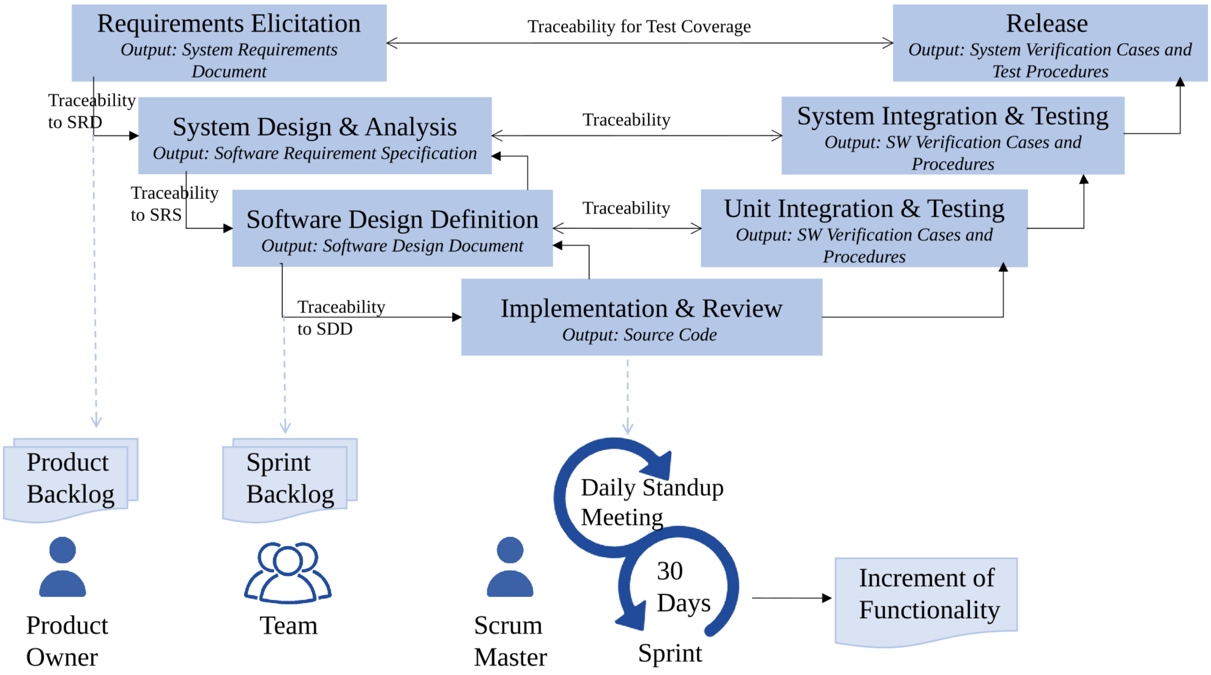

Figure S1. The AV model combines the V-model with Scrum artifacts while implementing traceability. It serves as a template for the process description of the app development and representation created with PPT in the style of McHugh et al.

We adjusted the AV model to the SLC guideline's requirements. Consequently, the SOP defines the SLC within a tailored V-model approach as the guideline recommends. It includes some Scrum artifacts like a sprint, the product and sprint backlog, and the definition of done. Moreover, it consists of a prototype. Figure S2 shows the used process, the deliverables of the activities and exercises, and the traceability between drafts and their consequences. The phases are not strictly sequential; returning to the previous step is always possible. An exception is the

[5] McHugh, Martin, Fergal McCaffery, and G Coady. 2015. 'Adopting Agile Practices When Developing Medical Device Software'. *Computer Engineering & Information Technology* 04 (02).

requirements elicitation, which must be done in advance for the high-level requirements. The problem-solving process is referenced.

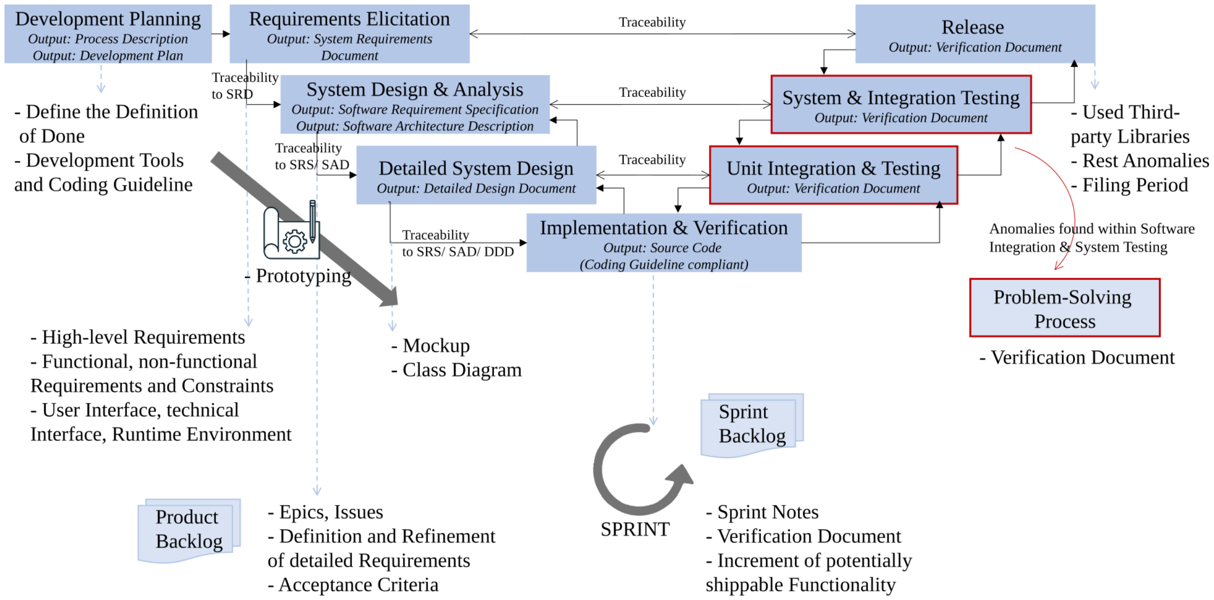

Figure S2. Process description of the app development within a tailored V-model, including Scrum artifacts and prototyping. The used processes and deliverable results of the activities and exercises, as well as the traceability between drafts and their results, are shown. One can always return to the prior phase, and only the high-level requirements must be defined in advance.

We adopted the guideline's definition of the problem-solving process and the integration and system testing, illustrated in Figure A2 and Figure A3. Since we will not use any legacy software within the app, it will be unnecessary to analyze or close the gaps between its documentation and the requirements by the IEC 62304.

The development plan is more detailed than the process description, adding concrete persons and tools to the process described as pictured in Figure S3. Since the developed application is simple, the development tools can be defined in advance, not having to suffice complex requirements. In more complex projects, it might be appropriate to represent them within the detailed system design phase. Since we did not want to develop exclusively for either iOS or Android, we used a cross-platform development framework. Flutter is a UI framework that Google released in 2018 for cross-platform mobile application development from a single codebase for both iOS and Android. Flutter does not replace traditional Android and Apple app models; Flutter functions as an app engine and introduces a new way of developing applications by writing them entirely using Flutter. The foundation of Flutter is Dart, an object-oriented language defined by classes and garbage collection. Widgets are the basic building blocks of Flutter's UI that consist of a widget tree. Stateful and stateless widgets are distinguished. Dynamic modification of stateful widgets is possible without reinitialization after they are built, whereas stateless widgets have no internal state that can be modified. Besides, the

development framework allows a "hot reload," which is very convenient for app development. If one changes the widget's color or size, a hot reload can be performed to instantly see the results on the simulator screen instead of re-running the whole project. Flutter can be used with any text editor. For this project, we chose Android Studio because of our personal experiences with the program and its support of Flutter editor plugins. Additionally, our supervisor suggested the usage of GitLab for source code management.

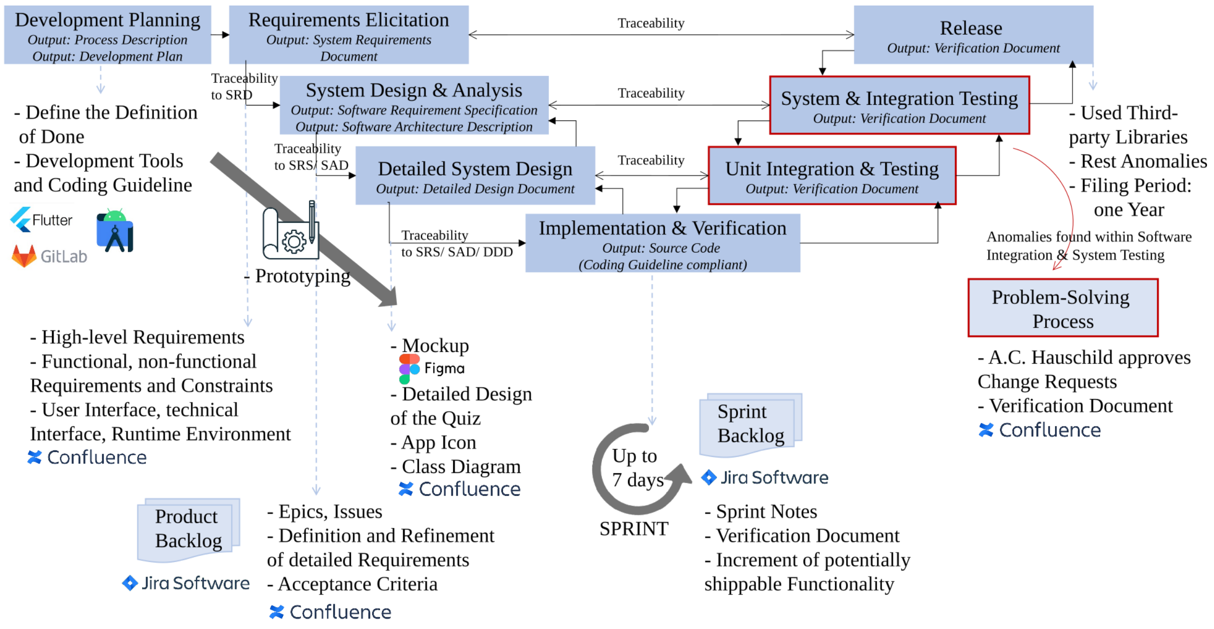

Figure S3. Concrete development plan of the diabetes app development project. It adds concrete persons and tools to the process description. Dr. Hauschild is the project supervisor, and Sabrina Holst is the developer. Flutter is the development framework installed within the text editor Android Studio. GitLab is used for source code management. The mockup is developed in Figma. Confluence contains all documentation, and project management is conducted in Jira, ensuring traceability and representation created with PPT.

It is impossible to distinguish system and software requirements within this project since the software is standalone. Consequently, the output "system requirement document" only refers to high-level requirements that can be defined before the development starts. They are highlighted as high-level requirements within the requirements specification in Confluence. Confluence serves as a wiki that contains all the documentation and enables mutual editing of documents. Using Confluence for documentation management is advantageous since the page history is stored and can be traced. One can publish the edits made with a version comment.

Consequently, all documents are under version control as demanded by the guideline. All requirements can be refined within the same document without losing the overview or the previous versions of the document. Refinement and

complementing of the high-level requirements and acceptance criteria occur within the system design and analysis. The main items are epics in Jira, a program for project and process traceability that supports the project organization in an agile context. The epics can be refined as tasks, bugs, or stories pooled as issues within Jira's manageable product and sprint backlog. During the implementation, one can move these issues from the product to the sprint backlog, define the sprint duration, start it formally, and document the issues' progress. One can reference epics and backlog items in the requirements specification, software architecture, and detailed design document in Confluence.

Furthermore, during system design and analysis, the prototyping begins. A strict differentiation between the prototype and the actual project is critical. However, prototypes help test ideas before they are officially implemented or changed within the project.

Within the detailed system design phase, the design of a mockup in Figma and more complex concepts of the architectural-defined modules are critical. Therefore, the quiz, as well as the app icon design, take place. Both software architecture description and the detailed design document are stored within the pages section in Confluence for better versioning control and traceability.

The actual implementation is organized within sprints. A sprint takes up to seven days, which is shorter than usual for Scrum since the app is a small development project, and we are the only developer. A requirement is fully implemented if it fulfills the predefined definition of done (DoD) and is consequently verified. The result is an increment of potentially shippable functionality, and a verification document is compiled. The sprint management is located in Jira, and the storage of sprint notes for each sprint and the verification document is in the pages section in Confluence. Traceability is ensured if the epics and issues in Jira are linked to the requirements in Confluence, and all requirements have unique IDs to use within the sprint notes, verification document, or a git commit to GitLab. The system design and analysis, detailed system design, implementation and verification, and the corresponding testing are repeated and refined throughout each sprint.

We import the anomalies found during the integration and the integration or software testing into the problem-solving process. Dr. Anne-Christin Hauschild, our supervisor, accepts change requests before changes within fully implemented and tested requirements may be made. Problem reports and change requests must be filed. Each test and verification must be documented within the verification documents section in Confluence. Though Dr. Hauschild monitors the project, we are the developer who makes all organizational, architectural, and programming decisions, which she approves before the actual implementation begins. The software is released in case all previous activities are concluded and their documentation is complete. The release verification document includes a list of software packages

used to ease future maintenance and all other anomalies. The project is stored for one year, and if the app is published or revised, this filing period must be extended.

All outputs within the process description are generated throughout the software development. The final versions of the software requirements specification, software architecture description, detailed design document and all verification documents, the coding guideline, change requests, and sprint notes are attached as PDF exports from Confluence within Appendix B. However, the process description and the development plan are not attached since they are introduced and explained within the thesis. Likewise, the code is not attached. It is only located within the GitLab repository.

After we drafted the development plan, we conceptualized the DoD, the coding guideline, and the rules for verification, as well as a structure for coding, testing, sprint notes, problem reports, and change requests. For developing the coding guideline, we used Flutter, Dart standards, and the style guide for Flutter on GitHub. The latest version can be found in Appendix B.5.

Each output must be verified throughout the development process or is a verification document. Table S2 shows the verification of the first outputs within the development plan. Since verification of the configuration elements is obligatory and must be planned, it is required at the latest within the implementation and verification phase. In case outputs are changed, they must be verified again. They are automatically reviewed during the implementation through the use of the DoD but should explicitly be reviewed again when the software is released.

Table S2. Verification of the outputs of the development plan.

System Requirements Document	Software Requirements Specification
- Must be derived from the stakeholder requirements<br>- May not contradict each other<br>- Must be consistent, unambiguous, clearly identifiable	- Software requirements implement the system requirements<br>- They may not contradict each other and must be consistent,<br>unambiguous and clearly identifiable<br>- They must be traceable to the system requirements or other sources<br>- Testing criteria must be derivable
**Software Architecture Description**	**Detailed Design Document**
- All system and software requirements are implemented<br>- The architecture must support the interfaces as well as SOUP items	- Implements does not contradict the software architecture.
**Source Code**	
- Coding guideline compliant<br>- Code is reviewed<br>(required by the DoD)	

The DoD checks if a requirement or related issues are fully implemented and can be tagged as completed within Confluence and Jira. It considers the verification of the outputs defined in Table S2. Within this project, a requirement is fully implemented, sufficing the DoD if:

- The code is coding guideline compliant.
- The code is consistent, unambiguous, and identifiable.
- A code review was conducted.
- All acceptance criteria are fulfilled.
- A build has been made and deployed in a testing environment.
- All necessary unit, integration, and system tests are passed and documented.
- Traceability to the requirements specification, epics, architecture, tests, and/or the detailed design document is implemented.
- The implementation does not contradict the architecture description or detailed design document.
- Sprint notes do exist.
- The documentation is complete.

This DoD is referenced within each verification document at the end of a sprint, and checkboxes are used to control the single requirements. An issue may only be removed from the product backlog and marked as done if it suffices the DoD. Documentation of a software unit's verification and all conducted tests is obligatory. Therefore, the outputs of the three final development activities are verification documents. Table S3 illustrates what a verification document must contain and which circumstances it must prove to verify a development activity correctly.

Table S3. Necessary content of the verification documents.

Unit Integration and Testing Verification Document	Software Release Verification Document
- Proves the integration of a software unit is realized according to an integration plan that can be derived from the software architecture<br>- Documentation should include: test case reference, result (pass/ fail), list of anomalies, software version, relevant tools, relevant software & hardware configuration, the person in charge, date	- The verification is complete, and all results are evaluated<br>- Rest anomalies are documented and evaluated<br>- All activities and tasks of the software development plan must be completed and documented<br>- Version of the released software<br>- Filing period for medical device software, all configuration elements, and the documentation<br>- How to ensure reliable delivery<br>- List of used third-party libraries
**Sprint Verification Document**	**System and Integration Testing Verification Document**
- The performed unit and system (integration) tests, considering their required documentation<br>- The DoD<br>- Open To-dos	- Documentation should include: test case reference, result (pass/fail), list of anomalies, software version, relevant tools, relevant software & hardware configuration, the person in charge, date

After planning the entire development process and integrating all the guideline's requirements, we set up the working framework. We installed all the necessary programs and created accounts for the required platforms. The setup of the development environment is explained within the verification document in Appendix B.7 to ensure the reproducibility of our results.

Software Requirement Analysis

Since the system requirements are derived from stakeholder requirements and determine the software requirements, it is essential to identify the project stakeholders first. Our stakeholders include our supervisor, Anne-Christin Hauschild, the biomedicine working group in general, and the FeatureCloud project for which we are writing the guideline. Potential users are interested in a healthy lifestyle and determining their diabetes risk because they have problems with healthy nutrition or getting enough physical activity. One could provide the app on a tablet to patients in waiting rooms of general practitioners to allow them to check their risk proactively. Consequently, the app is developed for medical laypersons. The high-level requirements are derived from conversations with our supervisor, her expectations, the app’s purpose, and other diabetes risk tests available online, such as the tests from the Deutsche Diabetes Stiftung or American Diabetes Association, as previously mentioned.

The app’s general purpose is to predict the user’s type 2 diabetes risk. Additionally, the app shall create early awareness of and attention to diabetes risk and help initialize life-saving lifestyle changes. Consequently, the user must answer a set of questions, the answers are stored, and the risk is calculated based on these answers. The critical variables and their thresholds must be identified, and an appropriate scoring system to predict the risk is necessary.

The first high-level requirements were defined in advance within Tables S4, S5, and S6 and were refined throughout the product development, as suggested by the guideline. The tables add to the first defined version of the requirements specification, and the system requirements document in Figure S3. It consists of three sections: functional requirements (Table S4), non-functional requirements (Table S5), and constraints (Table S6). The formulation template provided by the guideline is used, and the functional requirements are prioritized. They must be met before the product suffices the DoD and may be released. As the policy suggests, post-delivery maintenance, but more precisely, corrective maintenance is considered by providing the app developer’s contact information to the user.

Table S4. Functional high-level requirements.

	Requirement	User Story/System Requirement	Priority
1	High-level requirement	As a user, I want to get our diabetes type 2 risk.	Necessary
2	High-level requirement	As a user, I want to get further information about the test	Necessary
3	High-level requirement	As a user, I want to know how long the test takes	Nice to have
4	High-level requirement	As a user, I want to get explained what the calculated risk means and what consequences result.	Necessary
5	High-level requirement	As a user, I want to able to give our feedback or ask a question to the app's developer	Nice to have
6	High-level requirement	As a user, I want to use the app on a mobile device.	Necessary

Table S5. Non-functional requirements.

Quality Feature	Description
App's loading time	The app should load within 3 seconds
Reaction time	When navigating through the app a reaction should not take longer than one second.
Design	The design should be modern, authentic, and intuitive.
Screen's loading time	The transition between the screens should happen without delay to be as fluent as possible.
Maintainability	The software has to be maintainable to ease further development and use.
Specificity	The true negative rate measures the proportion of negatives that are correctly classified as not having the condition. The app should predict a low risk for at least 60 % of non-diabetic patients.
Sensitivity	The true positive rate measures the proportion of positive correctly classified as having the condition. The app should predict a high risk for at least 60% of the patients with diabetes or pre-diabetes.
Information Security	The user's information must be secure.
Unambiguity	The questions and answers must be unambiguous to ensure correct results.

Table S6. Constraints of the prototype app development project.

Constraint	Description
Laws	The app development is restricted by the Medical Device Regulation (MDR) and the IEC 62304 since it is medical device software (MDSW). The project is focused on the software life cycle. Consequently, other norms and laws for MDSW are not considered. Furthermore, the project is realized in academia considering the academic capabilities. The Software Life Cycle Guideline is used in order to assure
Costs	This project is a university project within the scope of a master thesis. Consequently, the software and programs must be free since no project budget is available.
Time	The whole thesis takes 6 months including 3 months of research and guideline development and 1 month of actual writing. The app development may not take longer than 2 months.

We evaluated several models that predict type 2 diabetes risk to find an appropriate scoring system. Several studies use a decision tree approach, a directed tree representing decision rules to classify or predict data. Habibi et al. developed a decision tree with good quality measures to predict if a person is diabetic or healthy [6]. However, the model requires the patients' systolic and diastolic blood pressure, which is difficult to measure for the app's target group. The same applies to the model by Huang et al., which integrates clinical and genetic features [7], both of which are impossible for medical laypersons to measure. Pei et al. developed a decision tree classifier with even higher quality measures, but the data basis was provided by a large Chinese hospital [8]. We expect the app's users to be German or European likely; therefore, it is unknown whether a model developed solely on Chinese patient data is comparable to German patient data.

Besides, Lui et al. rated a neural network approach with the highest precision power [9]. A neural network is a directed graph whose nodes are neurons that are activated, and the inputs are propagated through the network to be classified. A neural network approach is challenging to implement within an app based on Chinese patients' data. The FINDRISK is a popular screening tool developed in Helsinki using multivariate regression models that rely only on measurable factors with noninvasive methods [10]. Multivariate regression analyzes the relationship between one dependent and several independent variables, modeling that relationship through the usage of a regression function.

---

[6] Bellamy, Leanne, Juan-Pablo Casas, Aroon D Hingorani, and David Williams. 2009. 'Type 2 Diabetes Mellitus after Gestational Diabetes: A Systematic Review and Meta-Analysis'. *The Lancet* 373 (9677): 1773–79.

[7] Huang, Guan-Mau, Kai-Yao Huang, Tzong-Yi Lee, and Julia Tzu-Ya Weng. 2015. 'An Interpretable Rule-Based Diagnostic Classification of Diabetic Nephropathy among Type 2 Diabetes Patients'. *BMC Bioinformatics* 16 (1): S5.

[8] Pei, Dongmei, Yang Gong, Hong Kang, Chengpu Zhang, and Qiyong Guo. 2019. 'Accurate and Rapid Screening Model for Potential Diabetes Mellitus'. *BMC Medical Informatics and Decision Making* 19 (1): 41.

[9] Liu, Siyu, Yue Gao, Yuhang Shen, Min Zhang, Jingjing Li, and Pinghui Sun. 2019. 'Application of Three Statistical Models for Predicting the Risk of Diabetes'. *BMC Endocrine Disorders* 19 (1): 126.

[10] Schulze, Matthias B., Kurt Hoffmann, Heiner Boeing, Jakob Linseisen, Sabine Rohrmann, Matthias Möhlig, Andreas F.H. Pfeiffer, et al. 2007. 'An Accurate Risk Score Based on Anthropometric, Dietary, and Lifestyle Factors to Predict the Development of Type 2 Diabetes'. *Diabetes Care* 30 (3): 510–15.

The GDRS has a better predictive performance than the FINDRISK [11]. Consequently, we chose the GDRS, developed at the German Institute of Human Nutrition within a prospective cohort study with over 27,000 participants, as a scoring system. Three additional German study populations with more than 28,000 participants were used to validate the score. Each variable in the calculation of the GDRS was weighted through multivariate cox regression models [12]. They are regression models based on the immediate hazard, or the force of mortality, which is the risk that an event the subject did not have before will occur for the matter within a short time. Therefore, the variables strongly covarying with that event are identified [13]. First, one calculates a risk score and based on that score, the probability of developing diabetes during the following five years can be calculated.

The GDRS only includes noninvasive variables, which predict similarly or even better when compared to formerly published risk scores, including invasive measures. However, its results correlate with the OGTT. In 2014, the original GDRS from 2007 was adjusted based on user feedback to make it more understandable. The insights and scores of this updated GDRS are used within the following since its prediction accuracy is comparable to the original GDRS. Furthermore, the model focuses on established risk factors. Most elements are dietary and lifestyle-related, suggesting that their change reduces the risk of type 2 diabetes development. This does perfectly match the app's purpose. Moreover, the GDRS is suitable for the target group since the app is mainly developed for German and European users.

The non-functional requirements in Table S5 demand at least 60 % sensitivity and specificity for the app. The sensitivity is the true positive rate, measuring the proportion of positives correctly classified as having the condition. Specificity, the true negative rate, measures the proportion of negatives correctly classified as not having the disease. The GDRS's sensitivity for patients with an increased, high, and very high risk was 70 % and 100 %, and the specificity was 73 % and 64 % for prediabetes and diabetes, respectively. That does match the app's non-functional requirements.

Nevertheless, the accuracy of the risk estimation is limited by the self-reporting bias and a possible random error in the measurement of the variables. Physical activity is not measured objectively through heart rate, and smoking is a value-laden behavior that people tend to underreport. The GDRS is appropriate to identify individuals within the general population who have a high risk of developing type 2 diabetes.

After we chose the GDRS as a scoring system, the more detailed requirements elicitation was based on artifacts like system archeology and document analysis. The German Institute of Human Nutrition provides an online test for type 2 diabetes and a

---

[11] Trefflich, Iris, Carmen Jahn, and Franziska Jannasch. 2018. 'Application of Diabetes Risk Scores in Health Checkups. A Comparison of the German Diabetes Risk Score (GDRS) and FINDRISK Test'. *Ernahrungs Umschau*, no. 65(11) (November): 180–86.

[12] Mühlenbruch, Kristin, Hans-Georg Joost, and Matthias B. Schulze. 2014. 'Risk Prediction for Type 2 Diabetes in the German Population with the Updated German Diabetes Risk Score (GDRS).' *Ernahrungs Umschau*, no. 61 (6) (June): 90–93.

[13] Christensen, Erik. 1987. 'Multivariate Survival Analysis Using Cox's Regression Model'. *Hepatology* 7 (6): 1346–58.

questionnaire for patients and doctors. Primarily, we used the publications of Mühlenbruch *et al.* and Schulze *et al*. to derive the more detailed software requirements and architectural and detailed design decisions. Appendix B.1, the final version of the requirements specification can be consulted. It has been reviewed for completeness using the software product quality features by the ISO 25010.

Software Architecture and Software Design

The first fundamental architectural decision was whether the questions asked should be open- or closed-ended. Two calculation schemes of the GDRS exist, one for each type of data recording. Fixed answer options, instead of fields to type them in, reduce the risk of potential errors if users choose the wrong unit (for example, meters instead of centimeters) or have typos in their inputs. On the one hand, it minimizes the risk of misjudgments, specifying realistic ranges. On the other hand, that makes the data recording less precise. This is a disadvantage if further data investigation should be performed in the future based on the collected data. Since the app's primary purpose is accurate risk prediction and not data collection, we decided that closed-ended questions better suit the app's target group and minimize risk.

Although a simple mobile application's definition of a clear architecture is challenging, we chose a layered architecture approach. There are traditional architectural approaches for iOS and Android, but the app is developed for both platforms. Therefore, a more general attempt was necessary, drafted in Figure S4. The app is divided into three screens: the welcome, quiz, and result. A strict and clear differentiation is not possible. Still, within the presentation layer, the user interface is designed. Within the domain layer, the app coordination, logical decisions, and the movement and processing of data between the different layers are located. The infrastructure layer defines the data storage. Although there is no database in the first version of the software, its design allows eventual add-ons. The app has two primary interfaces - the user interface and the technical interface to the mobile device on which it operates. No other mobile device functionalities, like access to the camera or microphone, are needed; only the computational power, the disk space, and the touch screen functionality to allow the user to tap on the answers and buttons are needed. If a user taps the contact information, a mail draft automatically opens within their mail program. The flutter URL launcher automatically defines this interface; therefore, it does not need further specification. The architecture is a high-level definition and is incrementally refined throughout the implementation within the detailed design document.

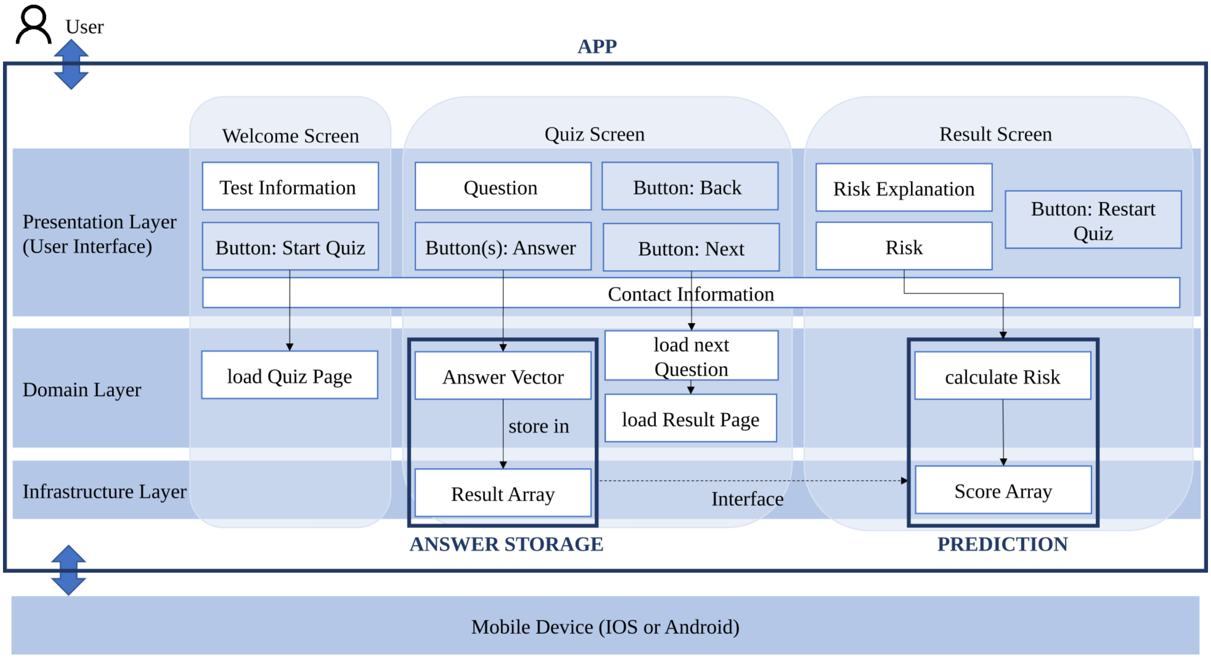

Figure S4. The layered software architecture of the diabetes app is divided into three screens: the welcome, the quiz, and the result screen. Within the presentation layer, the user interface is designed. Within the domain layer, the app coordination, logical decisions, and the movement and processing of data between the different layers are located. The infrastructure layer defines the data storage. The prediction of type 2 diabetes risk is divided into two parts: answer storage and prediction. Answer storage is the part collecting the values of the required variables. Within the prediction module, the actual risk score is calculated based on the inputs.

The type 2 diabetes risk prediction within the next five years, which is the software's main functionality, is divided into two parts: answer storage and prediction. Answer storage is the part collecting the values of the required variables. The actual risk score is calculated in the prediction module based on the inputs. Those two parts are isolated, only connecting through an interface. If the app is modified or extended, only the prediction module must be changed, for instance, to integrate another machine learning approach for prediction.

The detailed design document contains general design decisions like font size or color, the design of the app icon, the concrete realization of high-level requirement number six concerning corrective maintenance, a detailed quiz design, the result phrase, the project structure, and the class diagram. The final version can be found in Appendix B.3; the content was developed step-by-step throughout the implementation. In the following, we will explain the most fundamental design decisions.

The app mockup was designed and refined in Figma during the development process. It is pictured in Appendix B.4. Figma even allows for modeling the transitions between the different screens if the user performs specific actions;

therefore, we did not need any activity diagrams to model the order of the screens. Since they could easily be pictured, the mockup helped us discuss the first architectural decisions with our supervisor and potential stakeholders.

The quiz structure consists of questions, answers, answer vectors, their insertion index within the result array, and a score array drafted within Table S7. The questions, answers, one answer vector for each response, and its insertion index are stored within an array, a list object in the programming language Dart. This *kQuestions* list is located in the quiz service (see Figure S7). Each answer has a dedicated answer vector containing as many numbers as answer options exist. All numbers are equal to zero, except the position of the corresponding answer, which equals one. The dedicated answer vector is inserted within the result array at the insertion index position if the user chooses an answer option. The resulting array is illustrated in Figure S5. The sections reserved for the answers to a question within the result array are highlighted through colors in Figure S5. These colors can be found in Table S7, displaying the corresponding answers. The final result array only includes zeros and ones for those positions in which the user selected an answer. That eases the multiplication of the result array with the score array, adding only relevant scores. The risk is calculated based on this sum called the GDR score, or *riskScore* within Equation S1.

$$P(Diabates) = 1 - 0.99061^{\left(\frac{riskScore - 38.4558938)}{10}\right)}$$

Equation S1. Absolute diabetes risk calculation from Mühlenbruch et al.

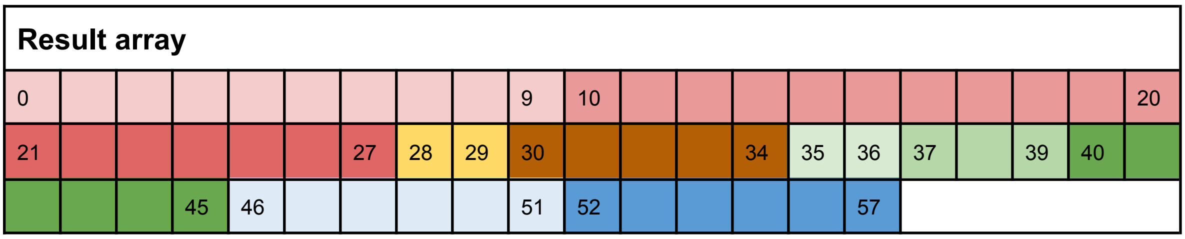

Figure S5. The resulting array within the answer storage module is in Figure S4. The answer vector dedicated to a particular answer, as represented in Table S7, is inserted at the insertion index position within the result array in case the user chooses a specific answer option. The sections reserved for answers to questions within the result array are highlighted through colors. These colors can be found in Table S7, displaying the belonging answers. For example, one answer vector from question one will be inserted between positions zero and nine (light pink) within the result array containing only zeros and a single one for the position in which the user selected an answer.

Table S7. Detailed design of the quiz.

Question/Answer	Result array insertion index	Answer vector	Score
What is your age in years?			
< 35	0	[1, 0, 0, 0, 0, 0, 0, 0, 0, 0]	0
35 - 39	0	[0, 1, 0, 0, 0, 0, 0, 0, 0, 0]	1
40 - 44	0	[0, 0, 1, 0, 0, 0, 0, 0, 0, 0]	4
45 - 49	0	[0, 0, 0, 1, 0, 0, 0, 0, 0, 0]	7
50 - 54	0	[0, 0, 0, 0, 1, 0, 0, 0, 0, 0]	10
55 -59	0	[0, 0, 0, 0, 0, 1, 0, 0, 0, 0]	13
60 - 64	0	[0, 0, 0, 0, 0, 0, 1, 0, 0, 0]	16
65 - 69	0	[0, 0, 0, 0, 0, 0, 0, 1, 0, 0]	19
70 - 74	0	[0, 0, 0, 0, 0, 0, 0, 0, 1, 0]	22
> 74	0	[0, 0, 0, 0, 0, 0, 0, 0, 0, 1]	25
What is your waist circumference in cm?			
< 75	10	[1, 0, 0, 0, 0, 0, 0, 0, 0, 0, 0]	0
75 - 79	10	[0, 1, 0, 0, 0, 0, 0, 0, 0, 0, 0]	5
80 - 84	10	[0, 0, 1, 0, 0, 0, 0, 0, 0, 0, 0]	8
85 - 89	10	[0, 0, 0, 1, 0, 0, 0, 0, 0, 0, 0]	12
90 - 94	10	[0, 0, 0, 0, 1, 0, 0, 0, 0, 0, 0]	16
95 - 99	10	[0, 0, 0, 0, 0, 1, 0, 0, 0, 0, 0]	20
100 - 104	10	[0, 0, 0, 0, 0, 0, 1, 0, 0, 0, 0]	24
105 - 109	10	[0, 0, 0, 0, 0, 0, 0, 1, 0, 0, 0]	28
110 - 114	10	[0, 0, 0, 0, 0, 0, 0, 0, 1, 0, 0]	32
115 - 119	10	[0, 0, 0, 0, 0, 0, 0, 0, 0, 1, 0]	36
≥ 120	10	[0, 0, 0, 0, 0, 0, 0, 0, 0, 0, 1]	40
What is your body height in cm?			
< 152	21	[1, 0, 0, 0, 0, 0, 0]	11
152 - 159	21	[0, 1, 0, 0, 0, 0, 0]	9
160 - 167	21	[0, 0, 1, 0, 0, 0, 0]	7
168 - 175	21	[0, 0, 0, 1, 0, 0, 0]	5
176 - 183	21	[0, 0, 0, 0, 1, 0, 0]	3

184 - 191	21	[0, 0, 0, 0, 0, 1, 0]	1
≥ 192	21	[0, 0, 0, 0, 0, 0, 1]	0
Have you ever been diagnosed with high blood pressure?			
No	28	[1, 0]	0
Yes	28	[0, 1]	5
What is your smoking status?			
Never smoked	30	[1, 0, 0, 0, 0]	0
Former smoker, < 20 cigarettes per day	30	[0, 1, 0, 0, 0]	1
Former smoker, 20 or more cigarettes per day	30	[0, 0, 1, 0, 0]	5
Current smoker, < 20 cigarettes per day	30	[0, 0, 0, 1, 0]	2
Current smoker, 20 or more cigarettes per day	30	[0, 0, 0, 0, 1]	8
How many hours a week are you physically active? Consider activities like sport, biking or gardening.			
Less than 5 hours a week	35	[1, 0]	1
5 or more hours a week	35	[0, 1]	0
How many cups of coffee do you consume?			
0 - 1 cups a day	37	[1, 0, 0]	3
2 - 5 cups a day	37	[0, 1, 0]	2
More than 5 cups a day	37	[0, 0, 1]	0
How high is your wholegrain (bread, muesli..) intake? Consider 1 portion as 1 slice or 3 tablespoons.			
0 portions a day	40	[1, 0, 0, 0, 0, 0]	5
1 portions a day	40	[0, 1, 0, 0, 0, 0]	4
2 portions a day	40	[0, 0, 1, 0, 0, 0]	3
3 portions a day	40	[0, 0, 0, 1, 0, 0]	2
4 portions a day	40	[0, 0, 0, 0, 1, 0]	1
More than 4 portions a day	40	[0, 0, 0, 0, 0, 1]	0
How much red meat (beef, pork, lamb..) do you eat?			
None or few	46	[1, 0, 0, 0, 0, 0]	0
1 - 2 times a week	46	[0, 1, 0, 0, 0, 0]	1
3 - 4 times a week	46	[0, 0, 1, 0, 0, 0]	3
5 - 6 times a week	46	[0, 0, 0, 1, 0, 0]	5

Daily consumption	46	[0, 0, 0, 0, 1, 0]	6
More than once a day	46	[0, 0, 0, 0, 0, 1]	8
What is your family history of diabetes?			
No history of diabetes in the family	52	[1, 0, 0, 0, 0, 0]	0
One parent with diabetes	52	[0, 1, 0, 0, 0, 0]	6
Both parents with diabetes	52	[0, 0, 1, 0, 0, 0]	11
At least one sibling with diabetes	52	[0, 0, 0, 1, 0, 0]	5
One parent and sibling(s) with diabetes	52	[0, 0, 0, 0, 1, 0]	6
Both parents and sibling(s) with diabetes	52	[0, 0, 0, 0, 0, 1]	11

The questions and answers must fulfill the non-functional requirement of unambiguity within the requirements specification in Appendix B.1. The GDRS asks for a history of hypertension, which is self-reported based on a prior medical assessment. Prevalent hypertension is defined as currently taking antihypertensive medication or having a systolic blood pressure of 140 or higher and a diastolic blood pressure of 90 or higher [14]. Since a medical layperson may not know what prevalent hypertension means, we simplified the formulation using the term “high blood pressure.” Furthermore, all possible answer scenarios must be included in the answer selection. Because of this, we decided to add two answer possibilities to question number ten concerning the family history of diabetes. The original GDRS only distinguishes between “No history of diabetes in the family,” “One parent with diabetes,” “Both parents with diabetes,” and “At least one sibling with diabetes.” A user could have one sibling and one or both parents with diabetes.

Consequently, we added “One parent and sibling(s) with diabetes” and “Both parents and sibling(s)” with diabetes to ensure that the users have those answer options and are not confused by which one they should select. The scores from one and both parents with diabetes are associated with these answer options since parents with diabetes have a higher impact than siblings. Additionally, the scoring scheme could not simply be changed because the impact of having both parent(s) and sibling(s) with diabetes was not considered within the original machine learning model. Overall, it was only a change within the user’s answer choices, not within the logic of the scores or result calculation.

[14] Egan, Brent M., und Yumin Zhao. „Different Definitions of Prevalent Hypertension Impact: The Clinical Epidemiology of Hypertension and Attainment of Healthy People Goals“. *The Journal of Clinical Hypertension* 15, Nr. 3 (26. Dezember 2012): 154–61.

riskScore		*riskPercent*	*resultPhrase*
> 105		100	Your risk of developing type 2 diabetes within the next 5 years is very high. It is possible that you already have diabetes. Urgently consult a doctor to get your blood glucose tested.
90		80	
81		50	
75		30	
70		20	Your risk of developing type 2 diabetes within the next 5 years is high. You should urgently consult a doctor to get your blood glucose tested.
63		10	Your risk of developing type 2 diabetes within the next 5 years is elevated. You should consult a doctor to get your blood glucose tested.
56		5	Your risk of developing type 2 diabetes within the next 5 years is low. You should watch your weight, eat healthy and increase your physical activity
46		2	Your risk of developing type 2 diabetes within the next 5 years is low.
39		1	
10			

Figure S6. The critical thresholds for the risk classification are stated on the result screen, as a resultPhrase. RiskScore, riskPercent, and resultPhrase are variables within the implementation of the resulting service (see Figure S7). The riskScore is the sum of the selected answer scores, and riskPercent is the percentage calculated based on the riskScore (see Equation 2). The resultPhrase is displayed on the result screen to explain the calculated riskPercent to the user.

The German Institute of Human Nutrition published critical thresholds for the risk classification, represented in Figure S6. The *resultPhrase* is the risk explanation item within the software architecture in Figure S4. A risk below 2 % is equal to low diabetes risk. A risk below 5 % is still low, but the person taking the test should watch their weight, eat healthily, and increase their physical activity since the diabetes risk increases with age. A risk between 5 % and 10 % is elevated, and a medical doctor recommends testing one's blood glucose. A high or very high diabetes risk starts at the threshold of 10 %, and especially with a risk over 30 %, it cannot be excluded that the person taking the test may already have diabetes.

Consequently, the app must give a diagnosis explaining the risk since a 10 % risk is classified as high, although that does not sound high for a layperson. Moreover, the percentage of the total *riskScore* is not proportional to the diabetes risk in percent. Half of the score (*riskScore* of 56) equals a *riskPercent* of around 5 %. Consequently, the user will receive the result in percent and an additional result phrase explaining it.

Within Figure S7, the project structure within Android Studio is shown. Our implementation follows the MVC pattern, differentiating between model, view, and controller as clearly as possible. The different pages are specified within the screens folder, predominantly the user interface and interaction design. We created the services to separate the user interface and the app's internal decision logic. The font resizing service adjusts the widgets' sizes depending on the device's screen size on which the app is displayed. The quiz service holds the *kQuestions* list, the app's central data structure, and the model within the MVC pattern. The result array is multiplied by the score array within the resulting service, and the risk percentage and the associated result phrase are calculated. This is the prediction item within the software architecture. The score array is part of the model, whereas the calculation is part of the controller within the MVC pattern. The other central part of the controller is the app state, which provides the different screens with information and contains the application logic that defines the system's reaction to a particular input. The app state is stored on which page the user selected which answer. The page equals the position within the *kQuestions* list (question one's position zero and, therefore, page zero).

Furthermore, the app state manages the result array. Consequently, the app state is the answer storage module defined within the software architecture. Suppose the user pushes a button or navigates on the screen, and the app state changes. The state management is realized through a provider pattern, and the navigation among the different screens is implemented through a navigator.

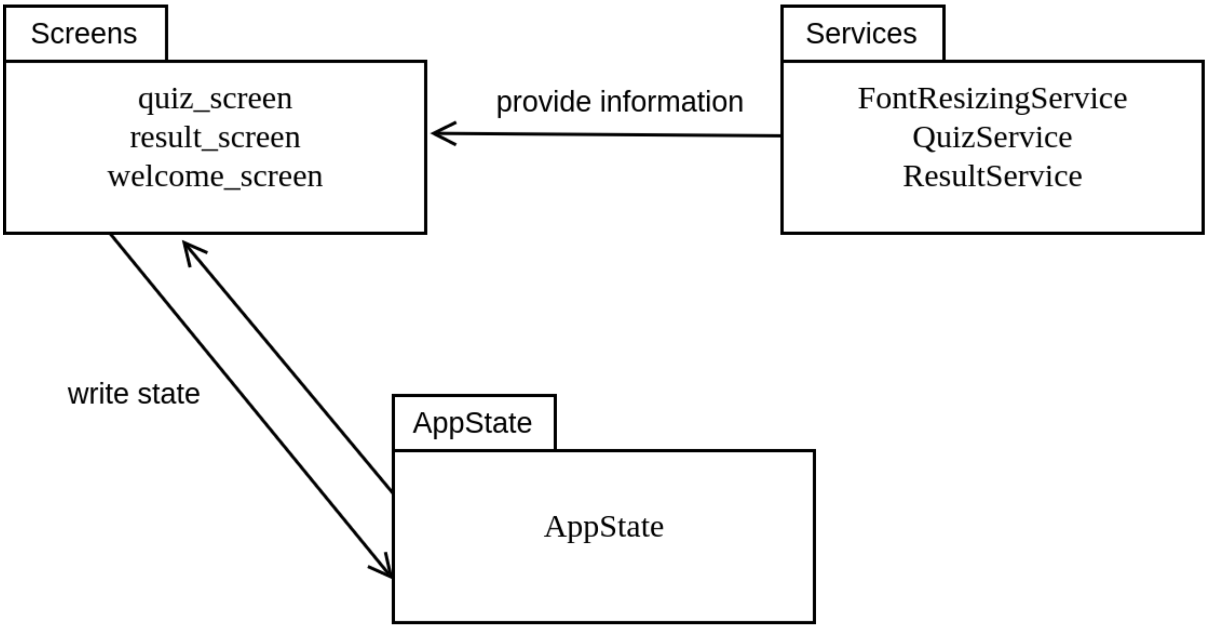

Figure S7. The detailed design of the project structure within Android Studio. The different pages are specified within the screens folder, predominantly the user interface and interaction design. The services separate the user interface and the app's internal decision logic. The app state provides the different screens with information and contains the application logic that defines the system's reaction to a specific input. The app state is stored on which page the user selected which answer. If the user pushes a button or navigates on the screen, the app state changes.

The more detailed class diagram can be found in Appendix B.3 in the detailed design. It is challenging to represent Flutter logic within a class diagram since the app structure is originally built of widgets within a widget tree. This is pictured within Figure A4.

Implementation, Testing and Verification

We divided the implementation into four sprints, which took between two and seven days each. For each sprint, sprint notes are filed within the sprint notes document in Appendix B.6. Each sprint is verified to ensure that the delivered code is DoD compliant, implements the guideline's verification requirements stated in Table S2, and is an increment of potentially shippable functionality. Consequently, the guideline suggests that the project is verified throughout the implementation process. The verification documents can be found in Appendix B.7.

We defined the quiz screen, the result screen, the welcome screen, and the app icon as epics within Jira. We added the services and app state throughout the development process to enable a better software architecture and differentiation between the user interface and decision logic. Within Figure A5, the epics and their associated issues are shown. Once we pushed a new project version on the GitLab

repository, we left a commit message specifying which problems have been implemented to ease traceability within the source code management. The final layout of the three screens is depicted in Figure S8.

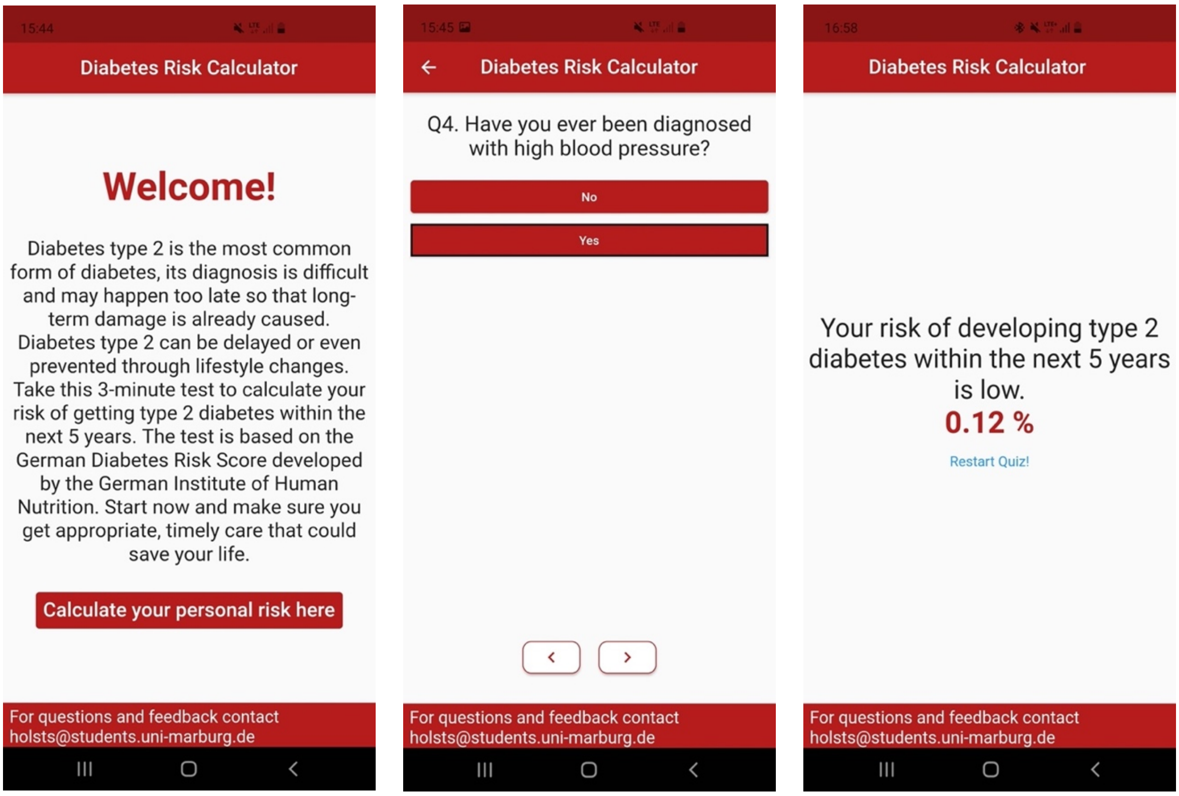

Figure S8. Final layout of the welcome screen, quiz screen, and result screen, screenshot of the Android test device.

Throughout the implementation process, we used a prototype to test our implementation ideas before making official decisions within the project.

Within the first sprint, we implemented the welcome screen. Then in the second sprint, the implementation of the quiz page occurred. Since the app architecture did not suffice the software architecture after the second sprint because the answer storage and prediction module were not isolated, we had to shift several issues to sprint three. Within this sprint, we implemented the navigator that connected the three screens and separated the resulting logic from the quiz page. By giving the app a state, we implemented the following navigation logic:

- If the user selects an answer, it is visually highlighted.
- Afterward, they are allowed to navigate forward when their selection is stored within the app state.
- They can also navigate back and change prior choices; the app state is dynamically updated.
- If they navigate back to the welcome screen using the app bar, the last question they worked on loads if the user restarts the quiz.

- After the last question is answered, they are forwarded to the result screen. They can only restart the quiz to assure a strict differentiation between the answer storage and the prediction module, as defined within the software architecture.

During the last sprint, we implemented the functionality behind the contact information. When the user taps on the bottom app bar, they are automatically forwarded to their mail program, and a draft with a predefined subject addressed to our email opens.

The final layout of all screens explaining the navigation logic is pictured within Figure A6. Within the requirements specification, the exact behavior of the software, the transition between the screens, as well as the functionality of the buttons are specified.

We adjusted the testing to the app’s process and the current level of integration as the guideline requires. Both a code review and a build were performed during each sprint for verification. Besides, we constantly repeated the tests conducted before, in case they were still relevant and were not replaced by automated ones. All tests must have been passed before one sprint was finished, and open issues were passed to the next sprint. The DoD compliance was the final step to verify each deliverable, summing up the tests conducted before and assuring the result adheres to the project’s requirements specification, software architecture, and detailed design. All verification documents containing a detailed description can be found in Appendix B.7.

During the first sprint, we performed a successful build on an Android virtual device (AVD) and the iOS simulator. Moreover, we checked the app’s user interface for a pleasant welcome page design on both screens to verify the sprint results. After having implemented the quiz page within the second sprint, we tested the correct implementation of the quiz logic. Therefore, we designed three manual test cases derived from the result phrase logic within Figure S6. They tested the result calculation, comparing the expected and the actual result for certain scores. We tested the restart quiz button to check if all variables were set to their default values after it was pressed, using print statements within the code. Thirdly, we tested the insertion of the answer vectors within the result array to ensure they were correctly implemented within the kQuestions list. The virtual device tests on the iOS simulator were successful, but the one on the AVD failed since the widgets exceeded the screen size. For that reason, we implemented a font resizing service, adjusting the widgets’ sizes to the device’s screen, and additionally implemented scrollable widgets in case the screen size is still exceeded.

To verify the results of the third sprint, we wrote automated test cases within the Android Studio project to test the modules defined in the software architecture as the guideline requires. The answer storage module is the app state, and we automated

test numbers two and three from sprint two to test it. The resetQuiz quiz function is the functionality behind the restart quiz button, and we wrote a test case checking if its use sets all variables to their default values as required. We modeled two test app states to test the borders of the inserted answer vectors within the result vector. One allows the user to always select the first answer, and one allows him to always choose the last one.

The second software module defined within the architecture is the prediction module, which is tested through result service testing. We defined one test case for each score border in Figure S6. We tried to vary the answers chosen to ensure that all scores within the score array were implemented correctly. Moreover, we tested the result phrase borders. Within the quiz service test, we checked if the questions were correctly stored within the kQuestions.

The best way to test mobile applications, especially if they do not have complex functionality or interfaces, is simply using them. Therefore, we tested the software on both iOS and Android physical devices. Afterward, we adjusted the screen design since it did not appear as appealing as expected on the physical devices we used. At that moment, we realized the challenge of building beautiful applications for different screen sizes. Afterward, we conducted a user test to get objective feedback on the app's functionalities. The user knew the software was not an officially registered MDSW and may only be used for test purposes. We observed his reactions while he calculated his risk but did not interfere. All functionalities seemed intuitive, and he understood all questions and answers. He even observed a typo within question number ten. Of course, for official alpha tests, one formally designs test cases for the user and collects much more data with several users in beta phase testing. For this reason, our test cannot be considered official usability testing, but since that is out of this thesis' scope, we thought one user's feedback, as well as that of Dr. Hauschild, to be sufficient.

To verify the increment of sprint four, in addition to the repeated tests, we tested the app on a virtual tablet iOS device to fulfill the acceptance criteria of requirement number five. Although the app development was focused on smartphones, we had to ensure that it also ran successfully on tablets. Finally, we installed the app on an Android device by providing its "app-release.apk" file on Google Drive.

Sprints and change requests were both verified. Change request one within Appendix B.8 addresses the design of the result since there were too many decimal places. It was initialized and approved by Dr. Hauschild. Consequently, we adjusted the percentage to two instead of three decimal places. Afterward, the build and all automated tests were repeated. The change request does not reference a problem report since it was not associated with a problem but only for aesthetic reasons. No problem occurred during the integration and system testing. Consequently, no problem report is filed within Appendix B, and the problem-solving process was not required.

Finally, the release verification document in Appendix B.7 states that the app is complete from the development point of view. It contains a list of the packages used to ease further maintenance and proves the verification required by the guideline and specified within Table S2 and Table S3. Additionally, it mentions the open tasks and next steps to enable a clean project handover in case someone else has refined the app.

Appendix

A.1 Additional Material

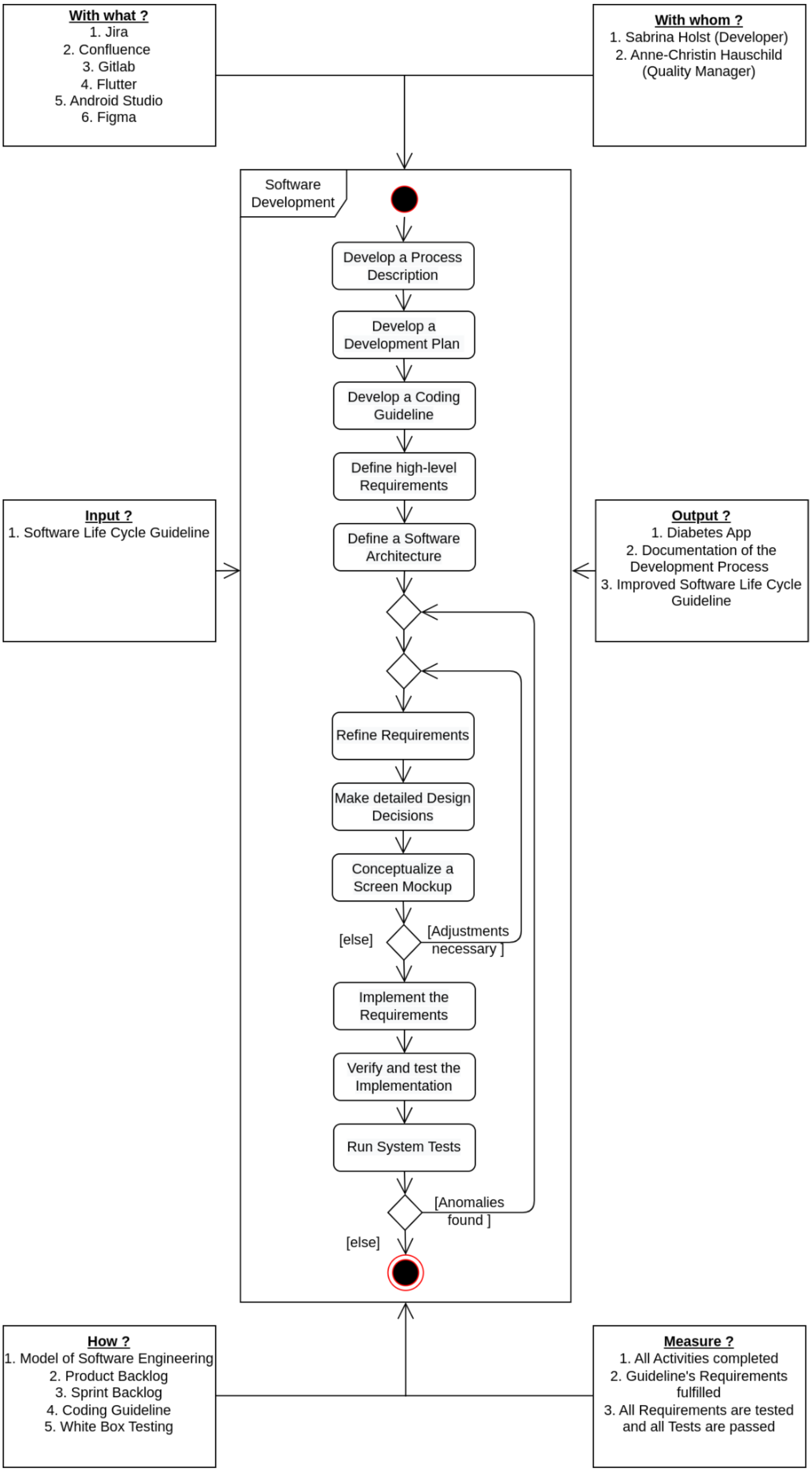

Figure A1. Turtle diagram as a tool to define relevant project processes.

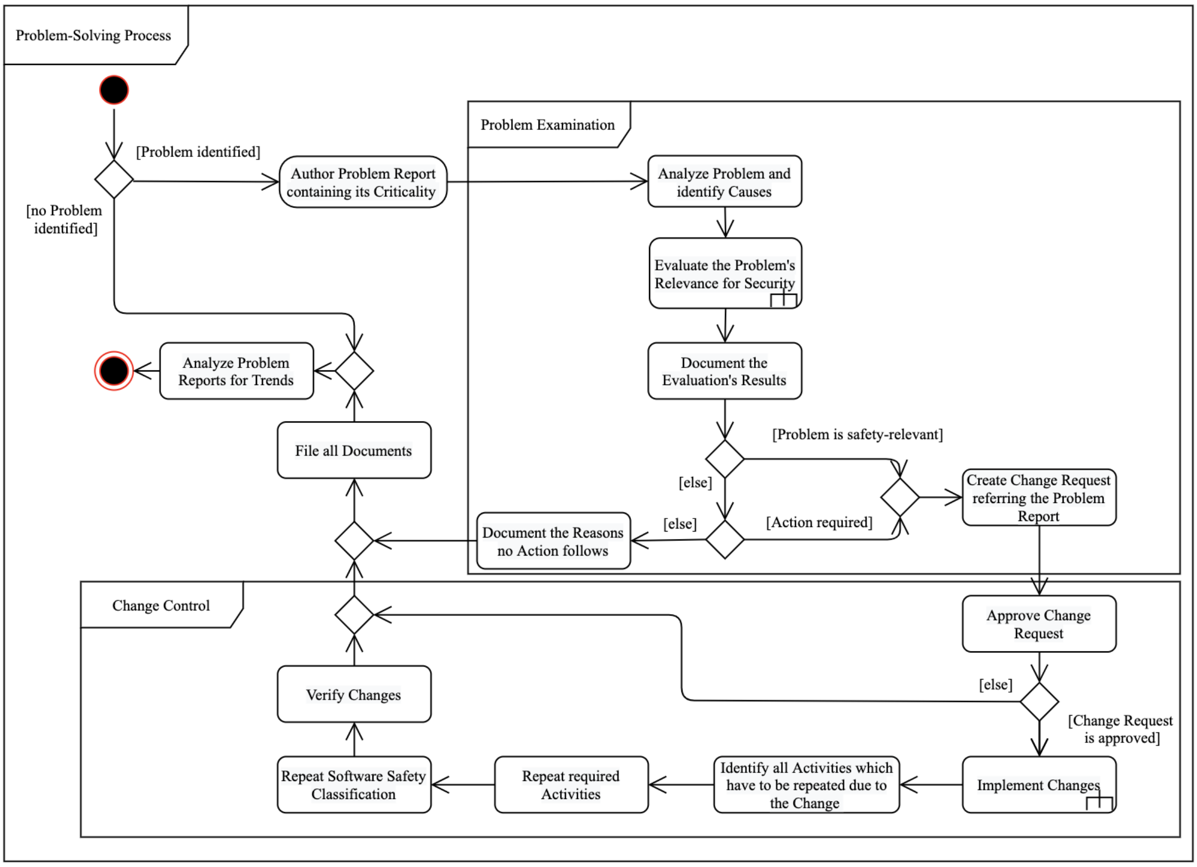

Figure A2. Activity diagram problem-solving process required by the IEC 62304.

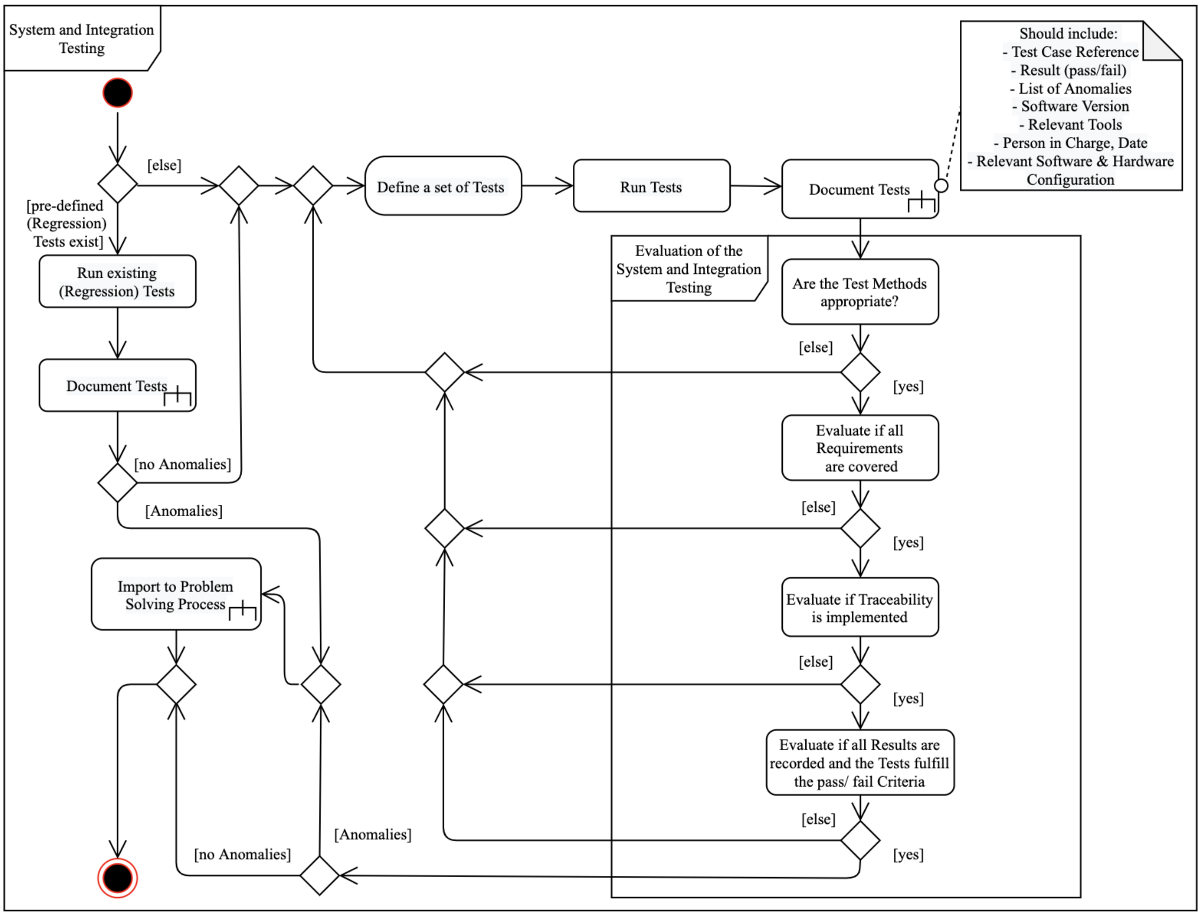

Figure A3. Activity diagram system and integration testing workflow required by the IEC 62304.

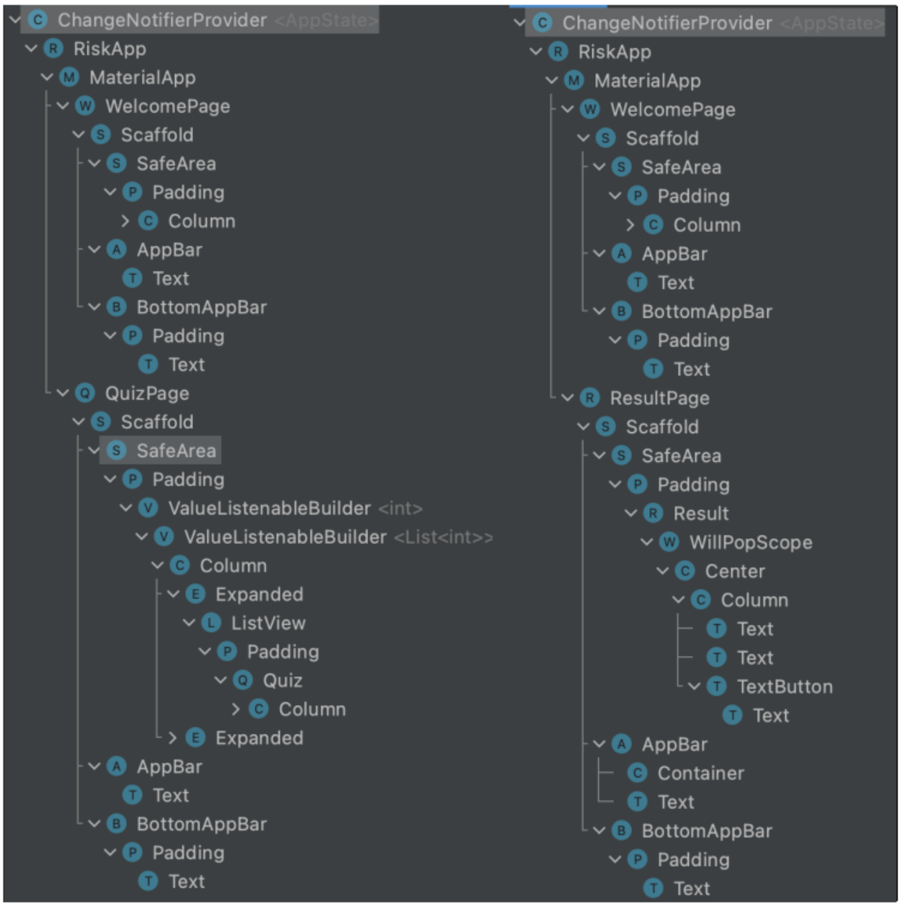

Figure A4. The Flutter project widget tree showing the widget hierarchy of the prototype app, screenshot of the Android Studio project.

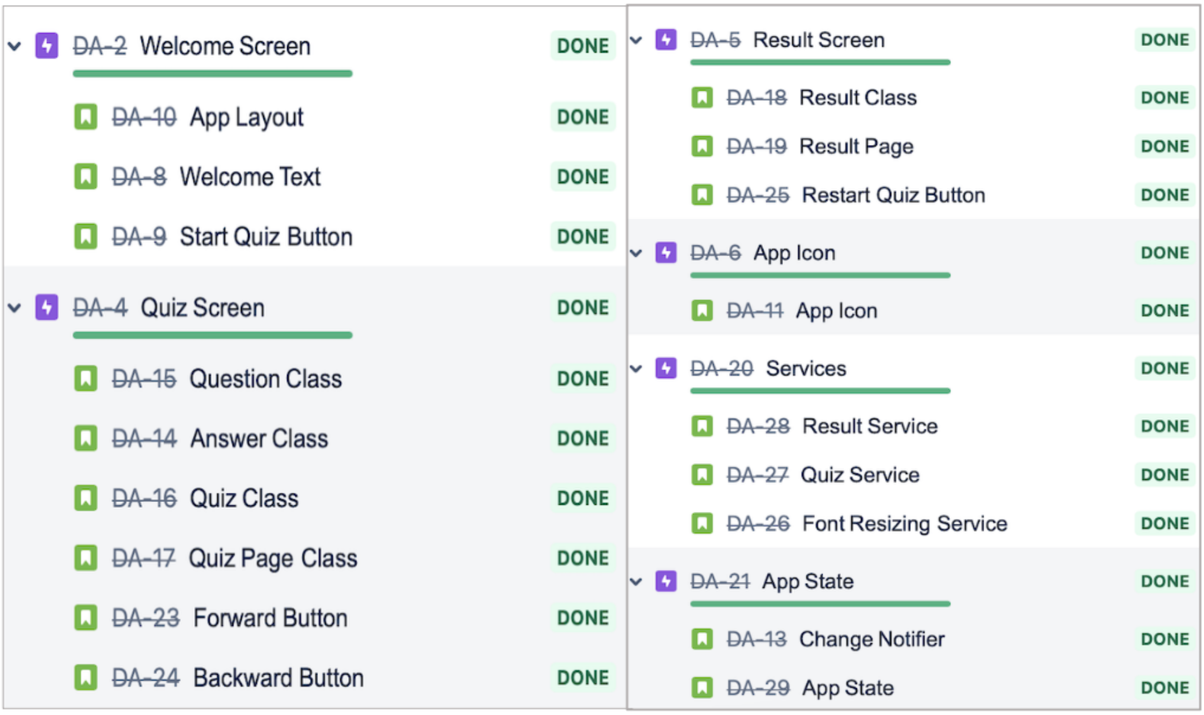

Figure A5. The project epics and associated issues, screenshot of the Jira roadmap.

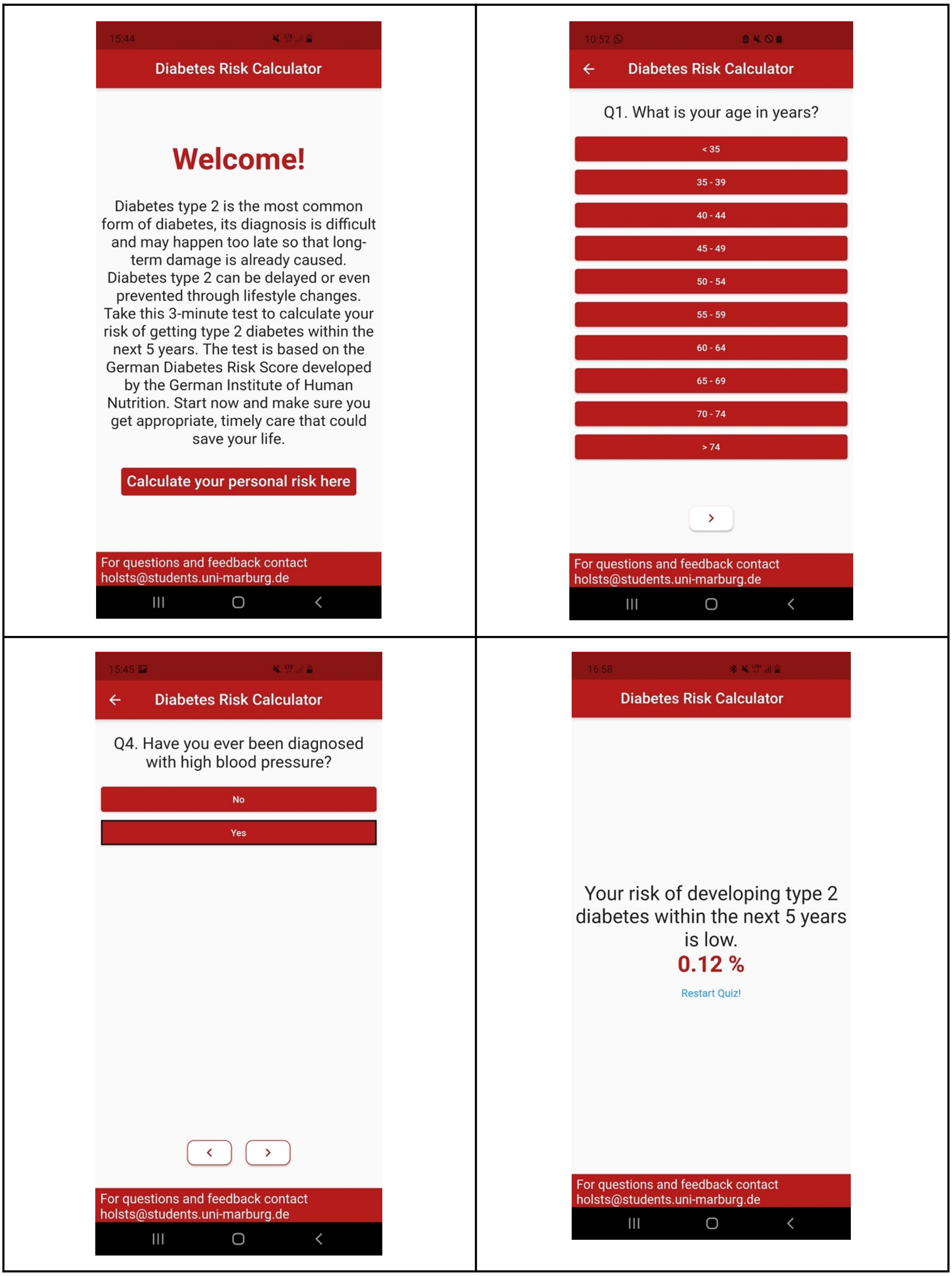

Figure A6. The final layout of the welcome screen, quiz screen, and result screen with arrows highlighting the navigation logic, screenshot of the physical Android test device.

B Outputs of the Project Development Plan

B.1 Requirement Specification

- Assumptions, Premises
- Functional Requirements
  - Detailed Specification and Acceptance Criteria
- Non-Functional Requirements
- Constraints

**Document State**	DONE
**Document Owner**	Sabrina Holst
**Spervisor**	Anne-Christin Hauschild

Assumptions, Premises

- The app's development follows the Software Life Cycle Guideline (https://docs.google.com/document/d/1p_aLBaF5aeA1kq39CuZ5NfCczAcVQtOxR3rsjujuqrQ/edit#). The app is a proof of concept for the guideline serving as a practical example demonstrating how the guideline should be applied and delivering helpful examples. It will be also used to improve the guideline.
- Template for user stories is: As a <user role>, I want <the action> in order to/ because <benefit>.
- Template for system requirements: Given <prerequisite> and <possible additional prerequisites> when <action> then <result/postcondition>.
- The detailed design of the screens is realized in Figma (https://www.figma.com/file/utWrtxftpaNxh4xQgapJZr/Diabetes-Risk-Calculation-App-Design?node-id=0%3A1).
- Stakeholder:
  - Dr. Hauschild
  - Data science in biomedicine working group
  - FeatureCloud
  - Potential users (patients within waiting rooms of general practitioners or people who are interested in a healthy lifestyle, or guess a diabetes risk, because they have problems with healthy nutrition or getting enough physical activity)
- Purpose: The app predicts the user's type 2 diabetes risk and shall arise early awareness and attention for diabets risk and help initializing live-saving lifestyle changes.

Functional Requirements

	Requirement	User Story / System Requirement	Priority	Notes
1	High-level Requirement<br><br>https://ecoup.atlassian.net/browse/DA-19<br><br>https://ecoup.atlassian.net/browse/DA-28<br><br>https://ecoup.atlassian.net/browse/DA-5	As a user, I want to get my diabetes type 2 risk.	NECESSARY	DONE
2	High-level Requirement<br><br>https://ecoup.atlassian.net/browse/DA-8<br><br>https://ecoup.atlassian.net/browse/DA-2	As a user, I want to get further information about the test.	NECESSARY	DONE
3	High-level Requirement<br><br>https://ecoup.atlassian.net/browse/DA-8<br><br>https://ecoup.atlassian.net/browse/DA-2	As a user, I want to know how long the test takes.	NICE TO HAVE	DONE
4	High-level Requirement<br><br>https://ecoup.atlassian.net/browse/DA-19<br><br>https://ecoup.atlassian.net/browse/DA-28 https://ecoup.atlassian.net/browse/DA-5	As a user, I want to get explained what the calculated risk means and what consequences result.	NECESSARY	DONE

5	High-level Requirement<br><br>https://ecoup.atlassian.net/browse/DA-22<br><br>https://ecoup.atlassian.net/browse/DA-30	As a user, I want to be able to give my feedback or ask a question to the app's developer.	NICE TO HAVE	DONE<br><br>Corrective maintenance
6	High-level Requirement<br><br>https://ecoup.atlassian.net/browse/DA-26	As a user, I want to use the app on a mobile device.	NECESSARY	DONE<br><br>The font resizing service was implemented to adjust the widget's sizes to different screen sizes.
7	https://ecoup.atlassian.net/browse/DA-6	When the user taps on the app icon, the app opens.	NECESSARY	DONE
8	https://ecoup.atlassian.net/browse/DA-2	After the app opened, the welcome screen is built.	NECESSARY	DONE
9	https://ecoup.atlassian.net/browse/DA-9<br><br>https://ecoup.atlassian.net/browse/DA-12	Given an opened welcome screen when the start quiz button is pressed then the quiz screen loads.	NECESSARY	DONE
10	https://ecoup.atlassian.net/browse/DA-14 https://ecoup.atlassian.net/browse/DA-16 https://ecoup.atlassian.net/browse/DA-17 https://ecoup.atlassian.net/browse/DA-15 https://ecoup.atlassian.net/browse/DA-27 https://ecoup.atlassian.net/browse/DA-4	As a user, I want to choose between different answers.	NECESSARY	DONE
11	https://ecoup.atlassian.net/browse/DA-24 https://ecoup.atlassian.net/browse/DA-13 https://ecoup.atlassian.net/browse/DA-29 https://ecoup.atlassian.net/browse/DA-21 https://ecoup.atlassian.net/browse/DA-17	As a user, I want to be able to navigate back in order to correct my answers.	NECESSARY	DONE
12	https://ecoup.atlassian.net/browse/DA-17 https://ecoup.atlassian.net/browse/DA-14	When the user taps on an answer then it has to be visually highlighted.	NICE TO HAVE	DONE
13	https://ecoup.atlassian.net/browse/DA-17 https://ecoup.atlassian.net/browse/DA-29 https://ecoup.atlassian.net/browse/DA-23	When the user has not selected an answer then he may not be able to navigate forward.	NECESSARY	DONE
14	https://ecoup.atlassian.net/browse/DA-17 https://ecoup.atlassian.net/browse/DA-29 https://ecoup.atlassian.net/browse/DA-24	When the user navigates back then he has to see which previous answer he selected and he must be able to change his selection.	NICE TO HAVE	DONE
15	https://ecoup.atlassian.net/browse/DA-12<br><br>https://ecoup.atlassian.net/browse/DA-17 https://ecoup.atlassian.net/browse/DA-5 https://ecoup.atlassian.net/browse/DA-23 https://ecoup.atlassian.net/browse/DA-29	Given the situation that the user selected the answer to the final question when he navigates forward then the result screen loads.	NECESSARY	DONE

Detailed Specification and Acceptance Criteria

The particular requirements and user stories are described in more detail and refined. Necessary steps for quality assurance, documentation, and testing can be derived.

US5 - As a user, I want to be able to give my feedback or ask a question to the app's developer.

Acceptence Criteria

- At all screens, the contact information are located within the bottom app bar.
- When the user taps on the app bar, he is forwarded to his standard mail program or he can choose which mail program he wants to use.
- When he chose a program, a mail draft with a pre-defined subject and recipient (the contact information) opens.

US6 - As a user, I want to use the app on a mobile device.

Acceptence Criteria

- The app is supposed to run on Android and IOS devices.
- In this context, mobile devices are defined as smartphones and tablets.

SR7 - When the user taps on the app icon, the app opens.

Acceptence Criteria

- The app icon matches the app's style (color scheme).
- The app icon looks health-related.

US8 - After the app opened, the welcome screen is built.

Acceptence Criteria

- When the user taps on the app icon the app opens and he sees a screen welcoming him.
- A text tells him what the test is about and how long it takes.
- The text has to inspire the user to take the test (stress topic's criticality).
- Below the text, the user sees a button:
  - The button says 'Calculate your personal risk here'.
  - The button is colorful (attracts attention).
  - By clicking on the button the user enters the quiz page.

US10 - As a user, I want to choose between different answers.

Acceptence Criteria

- The user looks at the quiz screen.
- At the top, he sees the question which has to be answered.
  - The question has to be unambiguous.
  - The question has to be easy to understand for medical laypersons.
  - The question has to contain the required measuring unit if necessary.
  - The question has to contain examples if necessary.
- Below the question, he sees the different answers he can choose between.
  - The answers have to be unambiguous.
  - The answers have to cover every possible case (lifestyle and body features).
  - The answers are kept as short and simple as possible.
- Only one answer can be chosen.

SR13 - When the user has not selected an answer then he may not be able to navigate forward.

Acceptence Criteria

- The user looks at the quiz screen.
- At the top, he sees the question which has to be answered.
- Below the question, he sees the different answers he can choose between.
- At the bottom of the page, he sees two buttons: (>) to go forward and (<) to go backward.
  - Given the situation that the user has not selected an answer, the button to go forward has to be disabled and therefore appears lighter than an enabled button.

SR14 - When the user navigates back then he has to see which previous answer he selected and he must be able to change his selection.

Acceptence Criteria

- The user looks at the quiz screen.
- At the top, he sees the question which has to be answered.
- Below the question, he sees the different answers he can choose between.
- At the bottom of the page, he sees two buttons: (>) to go forward and (<) to go backward.
  - Under the first question, the user may only see the button to go forward, since there is no prior question.
  - Backward navigation to the welcome screen is always possible using the app bar navigation.
    - Given the situation that the user has already answered some questions and then decides to navigate back to the welcome screen. If the user navigates back to the welcome screen and presses the button to calculate the risk (which is the only possibility to move forward again), he will be navigated to the last question he worked on before.
  - Backward navigation from the result screen to the quiz screen is not possible, since the software architecture demands a clear distinction between the answer storage and prediction module.
    - If the user restarts the quiz, he will be forwarded to the welcome screen.

B.2 Software Architecture Description

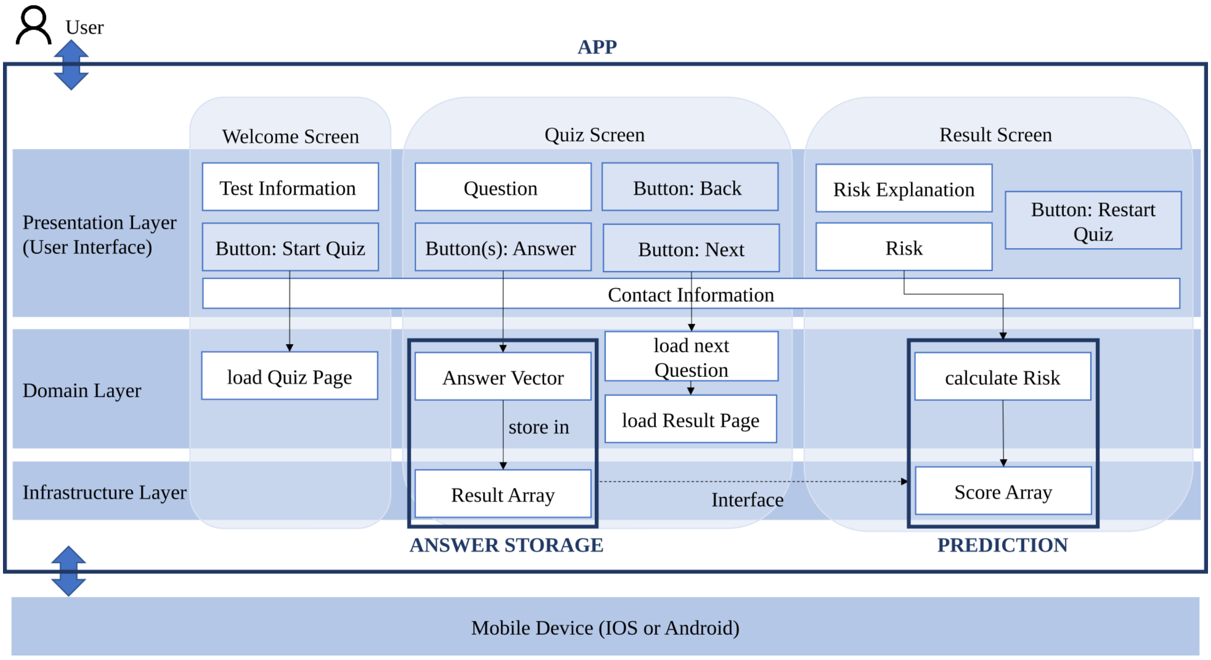

B.3 Software Architecture Description

- General Design Decisions
- App Icon
- Screen Design
- Software Maintenance
- Detailed Design of the Quiz
- Detailed Design of the Result Phrase
- Detailed Design of the Project Structure
- Class Diagram

General Design Decisions

- The main two colors of the app are red[900] and white.
- Information texts have a font size of 20 or 26.
- Headline/ highlighted text has a font size of 48.
- Questions have a font type of 26.
- Answers have a font type of 15.
- The contact information has a font type of 17.

This definition supports a consistent coding style, the https://ecoup.atlassian.net/browse/DA-26 adjusts the widget's sizes to the screen sizes of the device (to avoid exceedance).

App Icon

Requirements Specification SR-7

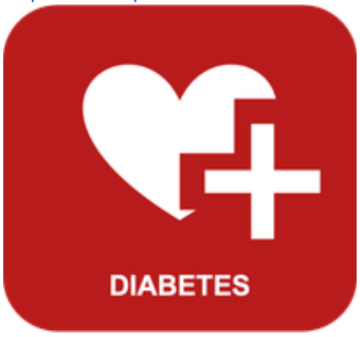

Screen Design

https://www.figma.com/file/utWrtxftpaNxh4xQgapJZr/Diabetes-Risk-Calculation-App-Design?node-id=0%3A1

Software Maintenance

Requirements Specification US-5

- For the app development, post-delivery maintenance was considered, more detailed corrective maintenance (see Software Architecture Description).
- The e-mail address of the developer is provided in order to provide an opportunity for the user to report observed errors or give feedback.
- '`For questions and feedback contact holsts@students.uni-marburg.de`' is displayed at the bottom of the screen.

Detailed Design of the Quiz

Requirements Specification US-10

Software Architecture Description Result Array, Score Array, Answer Vector, Question, and Answer

Question/ Answer	Result Array Insertion Index	Answer Vector	Score	Question/ Answer	Result Array Insertion Index	Answer Vector	Score	Result Array	
What is your age in years?				What is your smoking status?				0	35
< 35	0	[1, 0, 0, 0, 0, 0, 0, 0, 0, 0]	0	Never smoked	30	[1, 0, 0, 0]	0		36
35 - 39	0	[0, 1, 0, 0, 0, 0, 0, 0, 0, 0]	1	Former smoker, < 20 cigarettes per day	30	[0, 1, 0, 0]	1		37
40 - 44	0	[0, 0, 1, 0, 0, 0, 0, 0, 0, 0]	4	Former smoker, 20 or more cigarettes per day	30	[0, 0, 1, 0]	5		
45 - 49	0	[0, 0, 0, 1, 0, 0, 0, 0, 0, 0]	7	Current smoker, < 20 cigarettes per day	30	[0, 0, 0, 1, 0]	2		39
50 - 54	0	[0, 0, 0, 0, 1, 0, 0, 0, 0, 0]	10	Current smoker, 20 or more cigarettes per day	30	[0, 0, 0, 0, 1]	8		40
55 - 59	0	[0, 0, 0, 0, 0, 1, 0, 0, 0, 0]	13	How many hours a week are you physically active? Consider activities like sport, biking or gardening.					
60 - 64	0	[0, 0, 0, 0, 0, 0, 1, 0, 0, 0]	16	Less than 5 hours a week	35	[1, 0]	1		
65 - 69	0	[0, 0, 0, 0, 0, 0, 0, 1, 0, 0]	19	5 or more hours a week	35	[0, 1]	0		
70 - 74	0	[0, 0, 0, 0, 0, 0, 0, 0, 1, 0]	22	How many cups of coffee do you consume?				9	
> 74	0	[0, 0, 0, 0, 0, 0, 0, 0, 0, 1]	25	0 - 1 cups a day	37	[1, 0, 0]	3	10	45
What is your waist circumference in cm?'				2 - 5 cups a day	37	[0, 1, 0]	2		46
< 75	10	[1, 0, 0, 0, 0, 0, 0, 0, 0, 0, 0]	0	More than 5 cups a day	37	[0, 0, 1]	0		
75 - 79	10	[0, 1, 0, 0, 0, 0, 0, 0, 0, 0, 0]	4	How high is your wholegrain (bread, muesli..) intake? Consider 1 portion as 1 slice or 3 tablespoons.					
80 - 84	10	[0, 0, 1, 0, 0, 0, 0, 0, 0, 0, 0]	8	0 portions a day	40	[1, 0, 0, 0, 0, 0]	5		
85 - 89	10	[0, 0, 0, 1, 0, 0, 0, 0, 0, 0, 0]	12	1 portion a day	40	[0, 1, 0, 0, 0, 0]	4		
90 - 94	10	[0, 0, 0, 0, 1, 0, 0, 0, 0, 0, 0]	16	2 portions a day	40	[0, 0, 1, 0, 0, 0]	3		51
95 - 99	10	[0, 0, 0, 0, 0, 1, 0, 0, 0, 0, 0]	20	3 portions a day	40	[0, 0, 0, 1, 0, 0]	2		
100 - 104	10	[0, 0, 0, 0, 0, 0, 1, 0, 0, 0, 0]	24	4 portions a day	40	[0, 0, 0, 0, 1, 0]	1		
105 - 109	10	[0, 0, 0, 0, 0, 0, 0, 1, 0, 0, 0]	28	More than 4 portions a day	40	[0, 0, 0, 0, 0, 1]	0		
110 - 114	10	[0, 0, 0, 0, 0, 0, 0, 0, 1, 0, 0]	32	How much red meat (beef, pork, lamb..) do you eat?				20	
115 - 119	10	[0, 0, 0, 0, 0, 0, 0, 0, 0, 1, 0]	36	None or few	46	[1, 0, 0, 0, 0, 0]	0	21	
≥ 120	10	[0, 0, 0, 0, 0, 0, 0, 0, 0, 0, 1]	40	1 - 2 times a week	46	[0, 1, 0, 0, 0, 0]	1		57
What is your body height in cm?				3 - 4 times a week	46	[0, 0, 1, 0, 0, 0]	3		
< 152	21	[1, 0, 0, 0, 0, 0, 0]	11	5 - 6 times a week	46	[0, 0, 0, 1, 0, 0]	5		
152 - 159	21	[0, 1, 0, 0, 0, 0, 0]	9	Daily consumption	46	[0, 0, 0, 0, 1, 0]	6		
160 - 167	21	[0, 0, 1, 0, 0, 0, 0]	7	More than once a day	46	[0, 0, 0, 0, 0, 1]	8		
168 - 175	21	[0, 0, 0, 1, 0, 0, 0]	5	What is you family history of diabetes?				27	
176 - 183	21	[0, 0, 0, 0, 1, 0, 0]	3	No history of diabetes in the family	52	[1, 0, 0, 0, 0, 0]	0	28	
184 - 191	21	[0, 0, 0, 0, 0, 1, 0]	1	One parent with diabetes	52	[0, 1, 0, 0, 0, 0]	6	29	
≥ 192	21	[0, 0, 0, 0, 0, 0, 1]	0	Both parents with diabetes	52	[0, 0, 1, 0, 0, 0]	11	30	
Have you ever been diagnosed with high blood pressure?				At least one sibling with diabetes	52	[0, 0, 0, 1, 0, 0]	5		
No	28	[1, 0]	0	One parent and sibling(s) with diabetes	52	[0, 0, 0, 0, 1, 0]	6		
Yes	28	[0, 1]	5	Both parents and sibling(s) with diabetes	52	[0, 0, 0, 0, 0, 1]	11		
								34	

Detailed Design of the Result Phrase

Requirements Specification US-4

Software Architecture Description Risk Explanation

The resultArray is multiplied with the scoreArray, adding together only relevant scores (since the resultArray only consists of zeros and ones at the places where the user selected an answer). The sum (riskScore) is inserted in this Formula to calculate the absolute risk (riskPercent within the code):

$$\text{Absolute risk: } P(\text{Diabetes}) = 1 - 0.99061^{\exp\left(\frac{riskScore - 38.4558938}{10}\right)}$$

The resultPhrase is generated according to this risk:

riskScore		*riskPercent*	*resultPhrase*
> 105		100	Your risk of developing type 2 diabetes within the next 5 years is very high. It is possible that you already have diabetes. Urgently consult a doctor to get your blood glucose tested.
90		80	
81		50	
75		30	
70		20	Your risk of developing type 2 diabetes within the next 5 years is high. You should urgently consult a doctor to get your blood glucose tested.
63		10	
56		5	Your risk of developing type 2 diabetes within the next 5 years is elevated. You should consult a doctor to get your blood glucose tested.
46		2	Your risk of developing type 2 diabetes within the next 5 years is low. You should watch your weight, eat healthy and increase your physical activity
39		1	Your risk of developing type 2 diabetes within the next 5 years is low.
10			

Detailed Design of the Project Structure

This is the more detailed design of the project containing the main packages and classes. The state management is realized through the use of a provider pattern and the navigation between the different screens is implemented through a navigator.

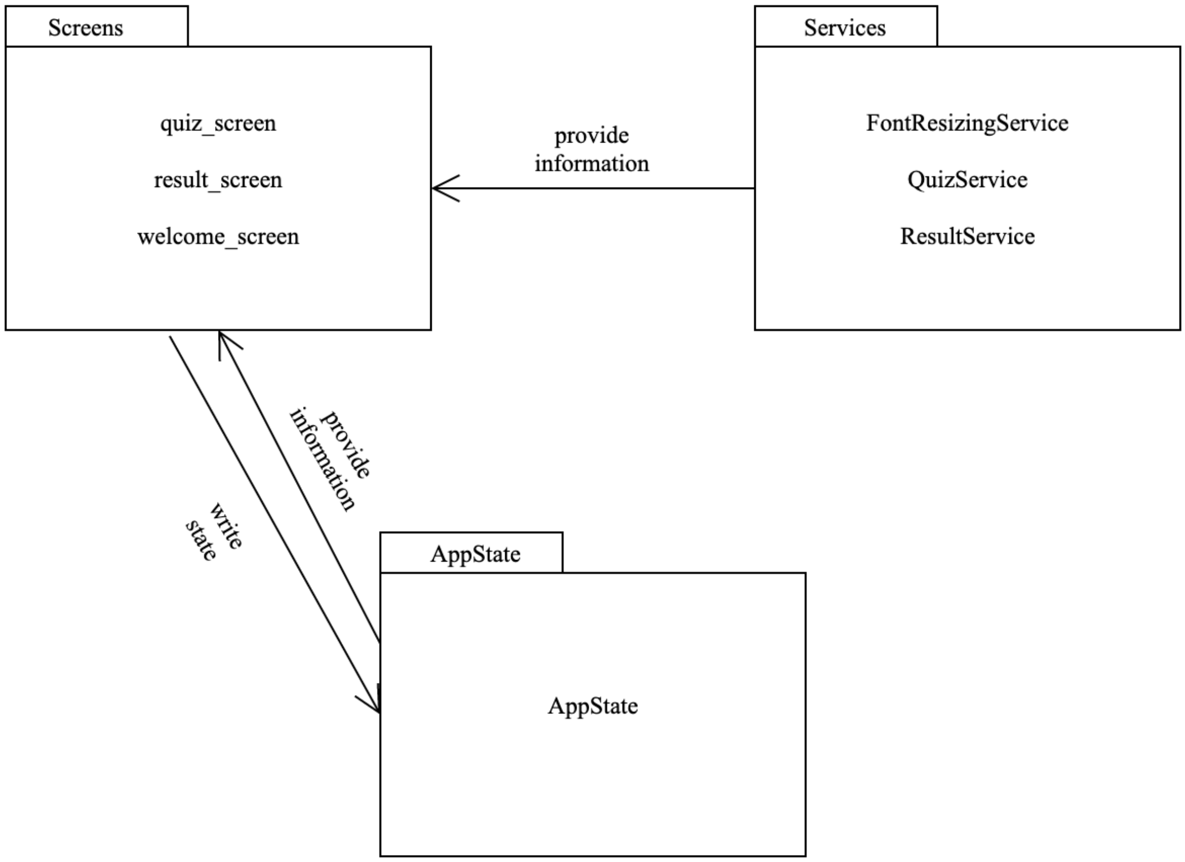

Class Diagram

The detailed design of the project structure is refined within this diagram. Only the main classes and their dependencies are shown. The navigator is implemented to change between the different screens, the provider for the app's state management, as well as the font resizing service to adjust the widget's sizes to fit different screens, are not shown. The focus is on the main classes which are important for the app's core structure. I tried to keep the flutter logic out of this diagram to achieve a distinction from the user interface view. Therefore, stateful and stateless widgets are not included as extensions within the diagram but only highlighted through two different shades of blue.

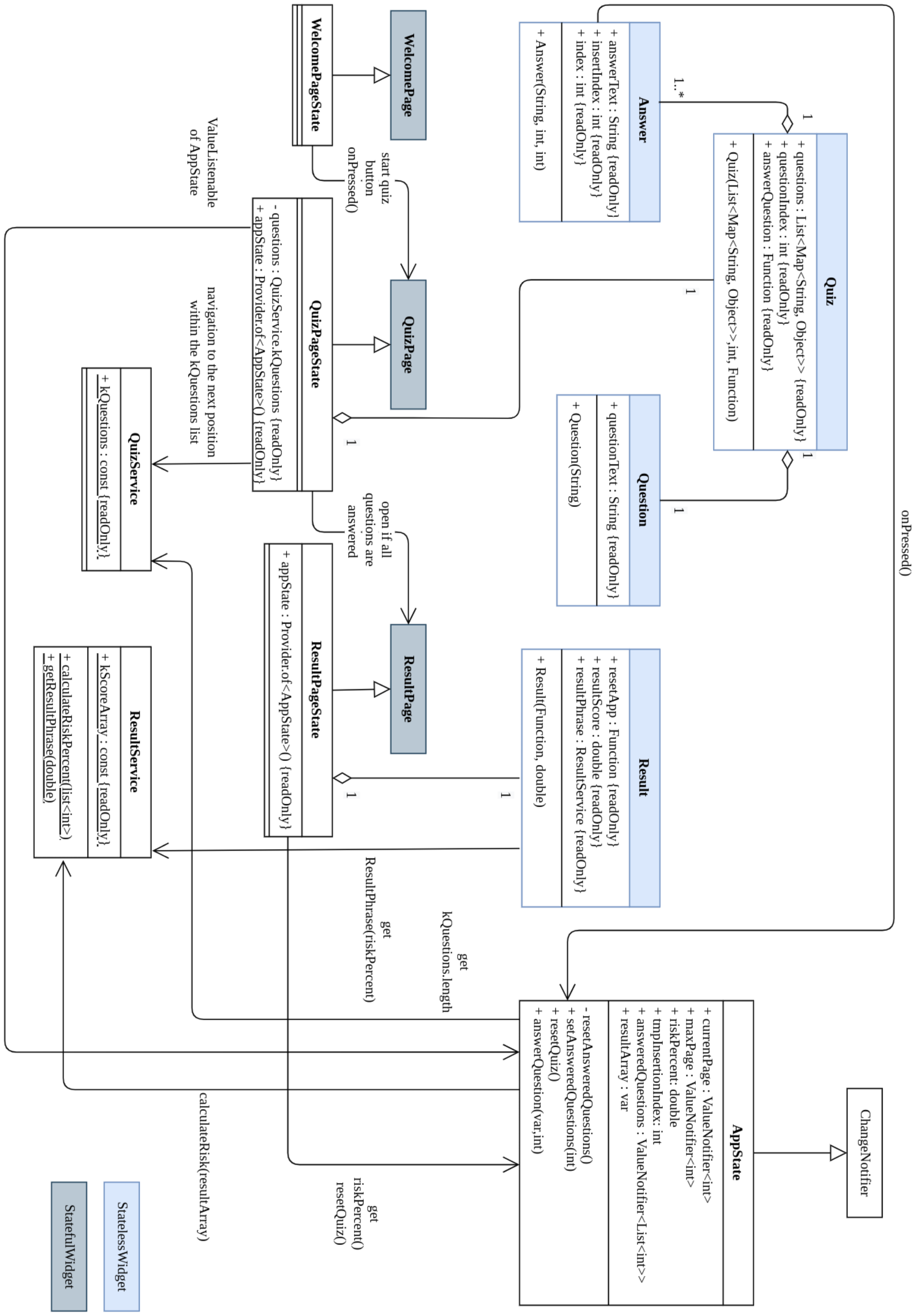

B.4 Mockup

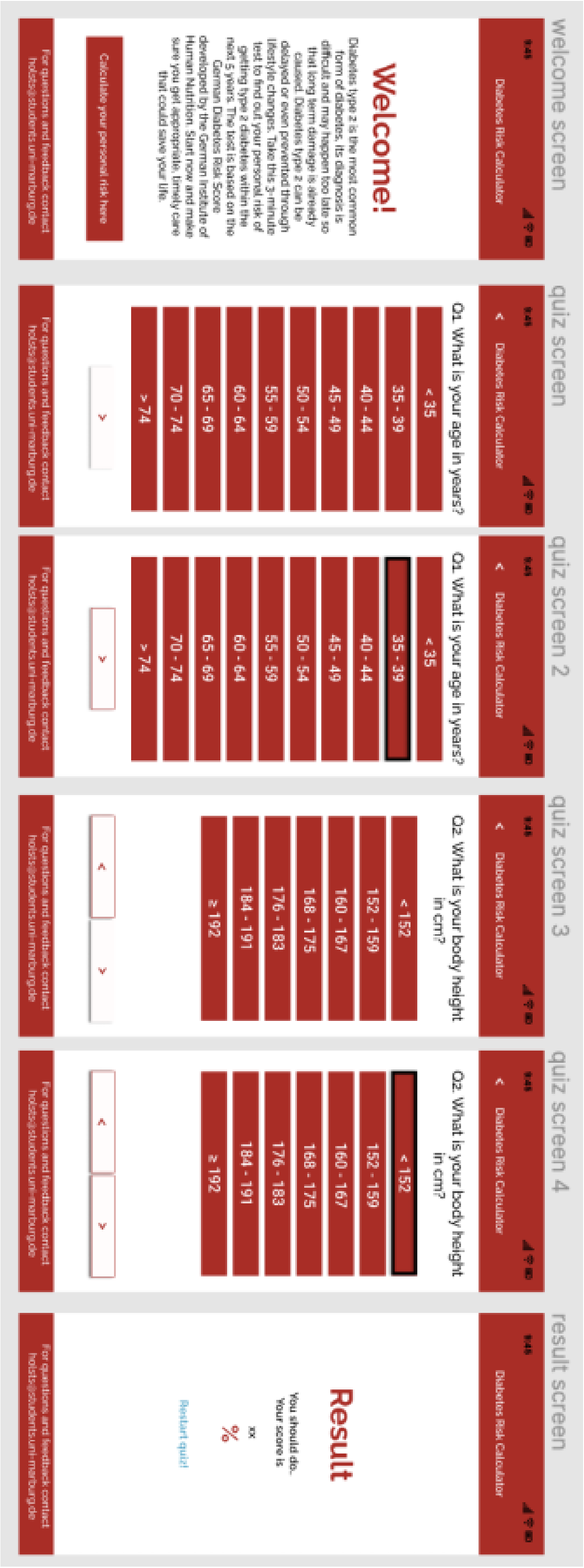

welcome screen
Diabetes Risk Calculator
Welcome!
Diabetes type 2 is the most common form of diabetes. Its diagnosis is difficult and may happen too late so that long term damage is already caused. Diabetes type 2 can be delayed or even prevented through lifestyle changes. Take this 3-minute test to find out your personal risk of getting type 2 diabetes within the next 5 years. The test is based on the German Diabetes Risk Score developed by the German Institute of Human Nutrition. Start now and make sure you get appropriate, timely care that could save your life.
Calculate your personal risk here
For questions and feedback contact holsts@students.uni-marburg.de
quiz screen
Q1. What is your age in years?
< 35
35 - 39
40 - 44
45 - 49
50 - 54
55 - 59
60 - 64
65 - 69
70 - 74
> 74
quiz screen 2
quiz screen 3
Q2. What is your body height in cm?
< 152
152 - 159
160 - 167
168 - 175
176 - 183
184 - 191
≥ 192
quiz screen 4
result screen
Result
You should do..
Your score is
xx
%
Restart quiz!

B.5 Coding Guideline

- Naming conventions
- Documentation
- To avoid exceptions or faults
- To ensure a good coding style and efficient runtime

The coding guideline was inspired by Flutter, Dart as well as the style guide for Flutter on GitHub:

https://github.com/flutter/flutter/wiki/Style-guide-for-Flutter-repo

https://dart.dev/guides/language/effective-dart/documentation#doc-comments

https://medium.com/flutter-community/flutter-best-practices-and-tips-7c2782c9ebb5

Naming conventions

- Spell correctly in US English
- Name classes, enums, typedefs, and extensions in `UpperCamelCase`
- Name variables, constants, parameters, and named parameters in `lowerCamelCase`
- Name libraries, packages, directories, and source files names in `snake_case(lowercase_with_underscores)`
- A correctly spelled word has no inner capitalization `scrollbar` but `appBar` since it's app bar
- Begin global constants with "k" `const double kFontSize = 1.5;`
- Avoid one-character names unless one character is idiomatic, prefer `index` over `i` but `x` over `horizontalAxis`

Documentation

- Format the comments like sentences and keep them as simple and short as possible
- Use the passive voice
- Begin comments with "//"
- Answer all questions that could arise by looking at the code
- The code's understandability is supported by the documentation
- Avoid useless documentation

```
// Bad: The background color is defined.

final Color backgroundColor;

// Good: The color filling the circle.

final Color backgroundColor;
```

- Comment empty closures to `setState()`

To avoid exceptions or faults

- Use `debugPrint()` instead of `print()`
- Use the `is` instead of the `as` cast operator

To ensure a good coding style and efficient runtime

- Make the state implementation of a widget private through the usage of underscores, so that it can only be instantiated by the corresponding StatefulWidget

```
class MyPage extends StatefulWidget {

  @override

  _MyPageState createState() => _MyPageState();

}
```

```
class _MyPageState extends State<MyPage> {

  @override

  Widget build(BuildContext context) {

    return Container();

  }

}
```

- If the value type of a class member is known it should be specified

`var item = 10;` use `int` instead of `var`

- If a sequence of operations is performed on an object use the Cascade “`..`” operator

```
// This isn't a good style.

var path = Path();

path.lineTo(0, size.height);

path.lineTo(size.width, size.height);

// This is better.

var path = Path();

..lineTo(0, size.height);

..lineTo(size.width, size.height);
```

- When `setState()` is called all descent widgets are rebuilt, so split the widget into small widgets so that the `setState()` call can exactly be placed at the subtree where the UI has to change

B.6 Sprint Notes

- Sprint One
- Sprint Two
- Sprint Three
- Sprint Four

Sprint One

21.05.-28.05.2021

https://ecoup.atlassian.net/browse/DA-6

https://ecoup.atlassian.net/browse/DA-8

https://ecoup.atlassian.net/browse/DA-10

https://ecoup.atlassian.net/browse/DA-9 - not finished within S1, implementation of onPressed() is missing

- 21.05 App icon design and implementation
- 23.05 Welcome text, contact information, and button text conception
- 24.05 Figma screen design and basic app design considerations (see Detailed Design Document)
- 25.05 Basic setup main.dart and welcomePage.dart implementation
- 26.05 Code review, testing (see https://ecoup.atlassian.net/wiki/spaces/DA/pages/370049134/Verification+Documents#Verification-Document-Sprint-1)

Sprint Two

31.05.-07.06.2021

https://ecoup.atlassian.net/browse/DA-14

https://ecoup.atlassian.net/browse/DA-15

https://ecoup.atlassian.net/browse/DA-16

https://ecoup.atlassian.net/browse/DA-17

https://ecoup.atlassian.net/browse/DA-18

https://ecoup.atlassian.net/browse/DA-19 - not finished within S2, separate from the quiz page

https://ecoup.atlassian.net/browse/DA-22

https://ecoup.atlassian.net/browse/DA-23 - not finished within S2, implementation of the functionality missing

https://ecoup.atlassian.net/browse/DA-24 - not finished within S2, implementation of the functionality missing

https://ecoup.atlassian.net/browse/DA-25

https://ecoup.atlassian.net/browse/DA-26- service is fully implemented, decide about usage within other screens

- The implementation of the start quiz button from sprint one will be moved to sprint three.
- 31.05 Detailed design of the quiz Detailed Design Document, started the implementation of DA14, DA15, and DA16 (the _questions list), implemented constant.dart (consistent color scheme) and text.dart (to shorten the code and edit the texts within one file)
  remark: changed name from _questions to kQuestions
- 01.06 Implemented DA18, refined DA17, detailed project structure Detailed Design Document
- 02.06 Better formatting of the result (round decimal number, percentage format), implemented DA19, DA23, DA24 (without the navigation functionality), first tests on the simulator were successful but the AVD test failed because the size of the widgets exceeded the screen's size (still after implementation of a ListView Widget to scroll through the answers);
  idea: write FontResizingService in order to adjust the widget's sizes to the screen size
- 04.06 Finished the implementation of the FontResizingService, code review, and testing (see https://ecoup.atlassian.net/wiki/spaces/DA/pages/370049134/Verification+Documents#Verification-Document-Sprint-2)
- 05.06 Refinements within the mockup design in https://www.figma.com/file/utWrtxftpaNxh4xQgapJZr/Diabetes-Risk-Calculation-App-Design?node-id=0%3A1, I realized that the required logic is too complex for further prototyping in Figma since the state cannot be modeled (the program does not know over which navigation you reached a certain page), further refinements will be realized within the

B.7 Verification Documents

- Requirements for the Verification Documents
- Definition of Done
- Android Studio Setup
- Verification Document Sprint 1
- Verification Document Sprint 2
- Verification Document Sprint 3
- Verification Document Sprint 4
- Verification Document Change Request 1
- Verification Document Software Release

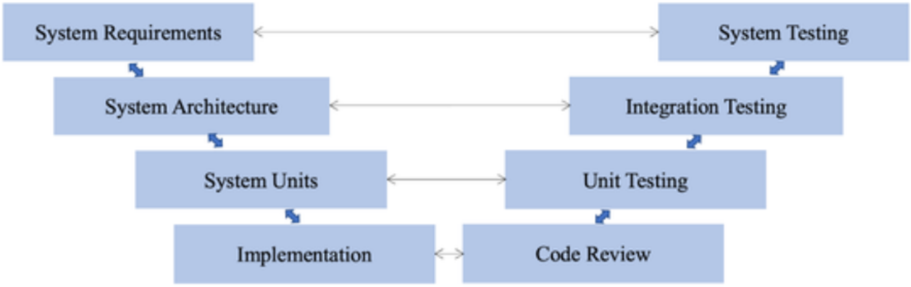

Requirements for the Verification Documents

Find the verfication document's requirements here

Unit integration and integration testing verification document:

- The integration of a software unit is realized according to an integration plan that can be derived from the software architecture.
- The documentation should include a test case reference, a result (pass/ fail), a list of anomalies, the software version, the relevant tools, the relevant software & hardware configuration, the person in charge, and the date.

System and integration testing verification document:

- The documentation should include a test case reference, a result (pass/ fail), a list of anomalies, the software version, the relevant tools, the relevant software & hardware configuration, the person in charge, and the date.

Sprint verification document

- The performed unit and system (integration) tests, considering their required documentation.
- The Definition of Done is filled in.
- Open To-dos are annotated.

Software release verification document:

- The verification is complete, and the results are evaluated.
- The rest anomalies are documented and evaluated.
- All activities and tasks of the software development plan must be completed and documented.
- The version of the released software.
- The medical device software, all configuration elements, and the documentation have to be filed for the whole lifetime of the software and as long as regulatory requirements demand.
- Documentation of how reliable delivery can be ensured.

Definition of Done

A requirement is fully implemented sufficing the definition of done (DoD) if:

- [ ] The code is coding guideline compliant.
- [ ] The code is consistent, unambiguous, and clearly identifiable.
- [ ] A code review was conducted.

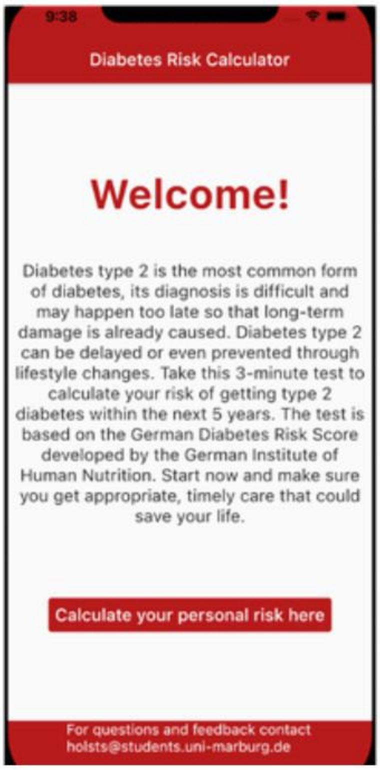

- The first android build failed.
    - The name app_icon.png did not match the AndroidManifest.xml.
    - The name was changed to ic_launcher.png.
- The second android build was successful.
    - There are no dart analysis issues or problems.

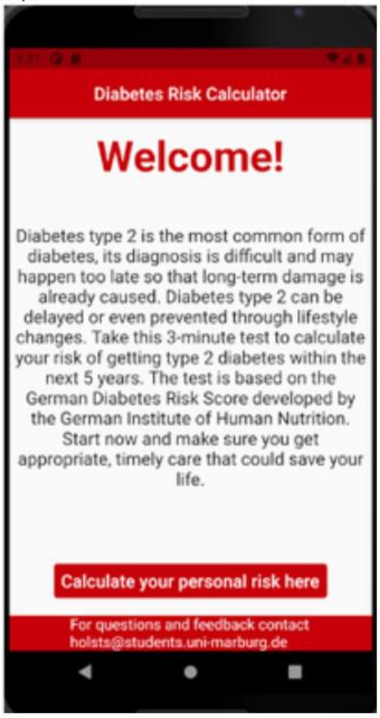

- The acceptance criteria for SR7 and US8 https://ecoup.atlassian.net/wiki/pages/resumedraft.action?draftId=336756793 and high-level requirements 2 and 3 are fulfilled.

- Open To-dos:
  - Implement the navigation to the quiz page https://ecoup.atlassian.net/browse/DA-9
- The requirements are DoD compliant:

Definition of Done

A requirement is fully implemented sufficing the definition of done if:

- [x] The code is coding guideline compliant.
- [x] The code is consistent, unambiguous, and clearly identifiable.
- [x] A code review was conducted.
- [x] All acceptance criteria are fulfilled.
- [x] A build has been made and deployed on a testing environment.
- [x] All necessary unit, integration, and system tests are passed and documented.
- [x] Traceability to the requirements specification, epics, architecture, tests, and/ or the detailed design document is implemented.
- [x] The implementation does not contradict the architecture description or detailed design document.
- [x] Sprint notes do exist.
- [x] The documentation is complete.

Verification Document Sprint 2

Software Version	1
Person in Charge	Sabrina Holst
Date	02.06.2021, 04.06.2021

- **Relevant Software Configuration and Tools:**
  Android Studio, Flutter, Dart, AVD, iOS Simulator (iPhone 12 pro Max) (for more details see https://ecoup.atlassian.net/wiki/spaces/DA/pages/370049134/Verification+Documents#Android-Studio-Setup)
- 02.06.2021: Conducted the first build on the Android virtual device.
  - The test failed because the widgets exceeded the size of the screen.
  - Idea: Implementation of service to adjust the sizes of the widgets based on the screen size (https://ecoup.atlassian.net/browse/DA-26).

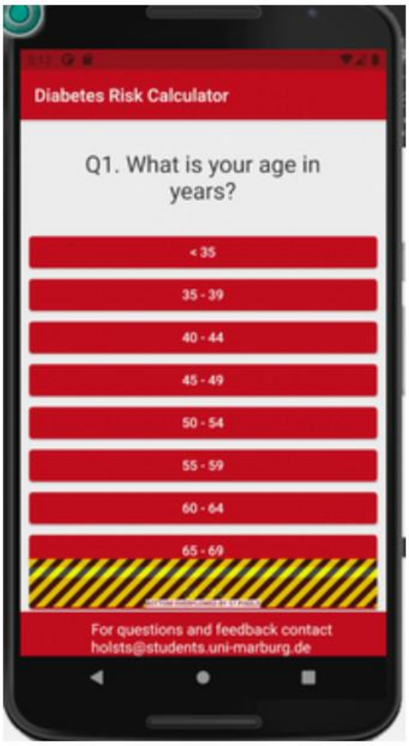

- 04.06.2021:
- The iOS Simulator build was successful.
  - There was one analysis issue: the unused import of one package which will be used in the further development.

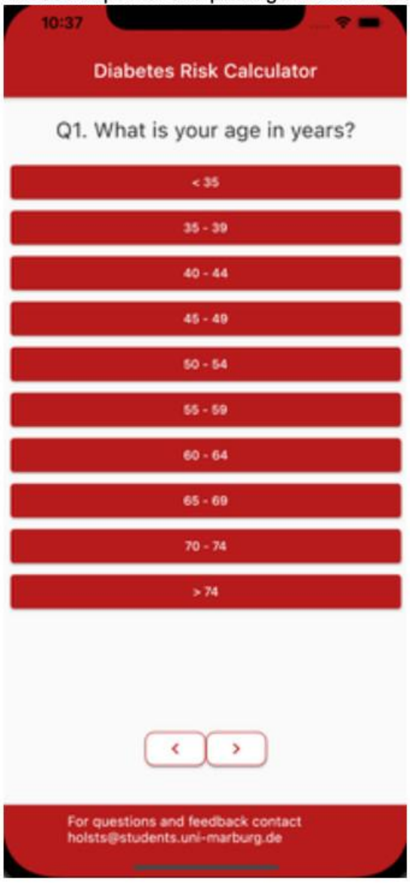

- The android build was successful.
  - There was one analysis issue: the unused import of one package which will be used in the further development.

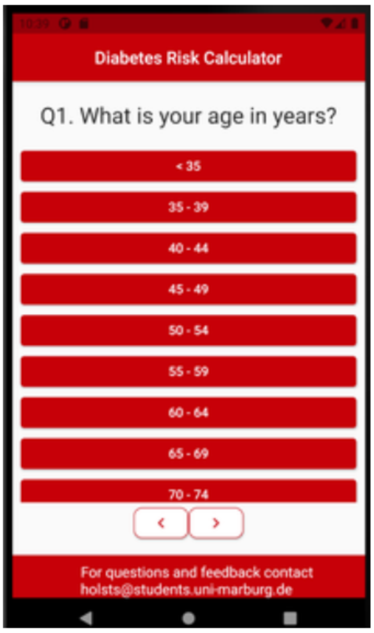

- Three manual test cases to test if the risk calculation is implemented correctly (1.1-1.3):
- **Test case 1.1:**
    - Expectation: If the user scores 39 points, the calculated risk is around 1 % (+-0.1 %).
    - Inputs:
        - age: 70-74 (score 22), waist circumference: < 75 (score 0), body heigt: 152-159 (score 9), high blood pressure: no (score 0), smoking status: current smoker < 20 (score 2), physical activity: 5 or more (score 0), coffee consumption: 0-1 cups (score 3), wholegrain intake: 3 portions (score 2), red meat: 1-2 times (score 1), family history: no (score 0)
          total score 39
    - Result: 0.991 % with the correct result phrase.
    - Test passed.
- **Test case 1.2:**
    - Expectation: If the user scores 90 points, the calculated risk is around 80 % (+-1 %).
    - Inputs:
        - age: 65-69 (score 19), waist circumference: >= 120 (score 40), body heigt: 176-183 (score 3), high blood pressure: yes (score 5), smoking status: former smoker 20 or more (score 5), physical activity: < 5 (score 1), coffee consumption: 2-5 cups (score 2), wholegrain intake: 4 portions (score 1), red meat: 3-4 times (score 3), family history: both parents (score 11)
          total score 90
    - Result: 80.485 % with the correct result phrase.
    - Test passed.
- **Test case 1.3:**
    - Expectation: If the user scores 70 points, the calculated risk is around 20 % (+-1 %).
    - Inputs:
        - age: 50-54 (score 10), waist circumference: 110-114 (score 32), body heigt: 160-167 (score 7), high blood pressure: yes (score 5), smoking status: current smoker 20 or more (score 8), physical activity: < 5 (score 1), coffee consumption: > 5 (score 0), wholegrain intake: > 4 (score 0), red meat: 1-2 times (score 1), family history: one parent and sibling(s) (score 6)
          total score 70
    - Result: 19.839 % with the correct result phrase.
    - Test passed.
- **Test case 2.1:**
    - Test the functionality of the restart quiz button.
    - Expectation: After the restart quiz button is pressed, the resultArray may only contain zeros.
    - Implementation: Usage of a print statement to see the resultArray.
    - Result: A resultArray containing only zeros.
    - Test passed.
- **Test case 2.2:**
    - Test the functionality of the restart quiz button.

- **Test case 2.3:**
  - Test the functionality of the restart quiz button.
  - The questionIndex of zero is implicitly tested because as soon as the restart quiz button is pressed, the first question is loaded again which equals the zero index within the kQuestions list.
  - Test passed.
- **Test case 3.1:**
  - Test if all indexes within the kQuestions list are set correctly.
  - Expectation: If the first answer is selected for each question, the ones within the resultArray have to be exactly at the positions defined within the kQuestions list (0, 10, 21, 28, 30, 35, 37, 40, 46, 52).
  - Implementation: Usage of a print statement to see the resultArray.
  - Result: A resultArray containing only zeros except from the ones at indexes 0, 10, 21, 28, 30, 35, 37, 40, 46, 52.
  - Test passed.
- **Test case 3.2:**
  - Test if all indexes within the kQuestions list are set correctly.
  - Expectation: If the last answer is selected for each question, the ones within the resultArray have to be at positions 9, 20, 27, 29, 34, 36, 39, 45, 51, 57.
  - Implementation: Usage of a print statement to see the resultArray.
  - Result: A resultArray containing only zeros except from the ones at indexes 9, 20, 27, 29, 34, 36, 39, 45, 51, 57.
  - Test passed.
- The acceptance criteria for US10 https://ecoup.atlassian.net/wiki/pages/resumedraft.action?draftId=336756793 are fulfilled as well as high-level requirements 1, 4, and 5.
- Open To-dos:
  - Implement the navigation to the quiz page https://ecoup.atlassian.net/browse/DA-9.
  - Isolate the result page from the quiz page and improve its layout (center) https://ecoup.atlassian.net/browse/DA-19.
  - Implement the functionalities of the https://ecoup.atlassian.net/browse/DA-23, and https://ecoup.atlassian.net/browse/DA-24, as well as the highlighting of the questions which are selected(https://ecoup.atlassian.net/wiki/pages/resumedraft.action?draftId=336756793 US/ SR12,13,14).
  - Consider implementation of the https://ecoup.atlassian.net/browse/DA-26 within other screens than only the quiz screen (final harmonization of the screens).
- The requirements are DoD compliant:
  - Exception 1: The result page layout does contradict the design in https://www.figma.com/file/utWrtxftpaNxh4xQgapJZr/Diabetes-Risk-Calculation-App-Design?node-id=0%3A1 but this will be fixed within the next sprint when the https://ecoup.atlassian.net/browse/DA-19 is finalized (the text is not centered within the screen).
  - Exception 2: The answer storage and the prediction module are not isolated from each other like demanded within the Software Architecture Description. This will be realized through the implementation of the services (https://ecoup.atlassian.net/browse/DA-20) within sprint three.

Definition of Done

A requirement is fully implemented sufficing the definition of done if:

- [x] The code is coding guideline compliant.
- [x] The code is consistent, unambiguous, and clearly identifiable.
- [x] A code review was conducted.
- [x] All acceptance criteria are fulfilled.
- [x] A build has been made and deployed on a testing environment.
- [x] All necessary unit, integration, and system tests are passed and documented.
- [x] Traceability to the requirements specification, epics, architecture, tests, and/ or the detailed design document is implemented.
- [ ] The implementation does not contradict the architecture description or detailed design document.
- [x] Sprint notes do exist.
- [x] The documentation is complete.

Software Version	1
Person in Charge	Sabrina Holst
Date	10.06.2021, 14.06.2021

- **Relevant Software Configuration and Tools:**
  Android Studio, Flutter, Dart, XCode, AVD, iOS Simulator (iPhone 12 pro Max) (for more details see https://ecoup.atlassian.net/wiki/spaces/DA/pages/370049134/Verification+Documents#Android-Studio-Setup)

  Test devices: iPhone 8 plus (software version 14.4.2), Samsung Galaxy S10+ (Android version 11)
- 10.06.2021: Succesful build on the Android virtual device and the iOS Simulator.
  - There are no analysis issues and only two pre-defined To-dos for Android developers (ID and signing config) which are not relevant until the app is actually released.
- Automated test cases are written within the project's test directory.
- The app state, the quiz service, and the result service are tested to test especially the components defined within the architecture (prediction module and answer storage module).
  - The app state is basically the answer storage module (see Software Architecture Description).
    - It is tested if the resultArray, as well as all insertion indexes within the quiz service, are defined correctly. Consequently, the edges are tested and the use case that the user always selects the first and always selects the last answer are modeled. That tests https://ecoup.atlassian.net/wiki/spaces/DA/pages/370016307/Detailed+Design+Document#Detailed-Design-of-the-Quiz.
  - The result service is basically the prediction module (see Software Architecture Description).
    - For all amounts of points within the https://ecoup.atlassian.net/wiki/spaces/DA/pages/370016307/Detailed+Design+Document#Detailed-Design-of-the-Result-Phrase the risk percent score calculation is tested and each condition within the loop for the result phrase is entered once (to reach full path coverage).
  - All test cases are passed.
- The use case tests from sprint 2 are repeated.
  - They are all passed.
- The software tests have to be performed on the actual physical devices.
- iOS device test:
  - The XCode version (12.5) is compatible with the iOS version on my mobile device (14.4.2).
  - The build was successful.
  - While running on the iPhone the layout of the screen did not look good.

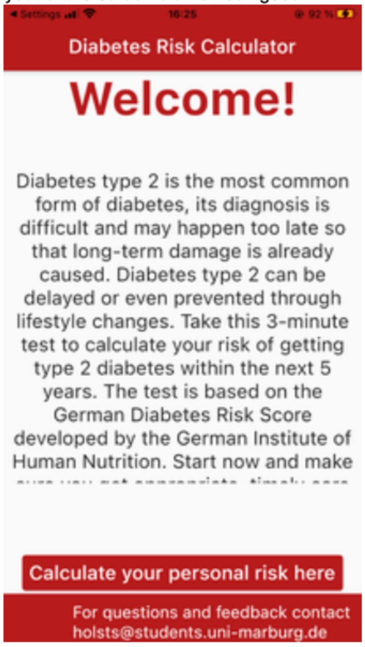

- I removed the scrollable around the welcome text and adjusted the sizes of the boxes.
- I adjusted the position of the contact information within the bottom app bar.
- I rebuild the APK and used the simulator and AVD again, the layout worked for both.

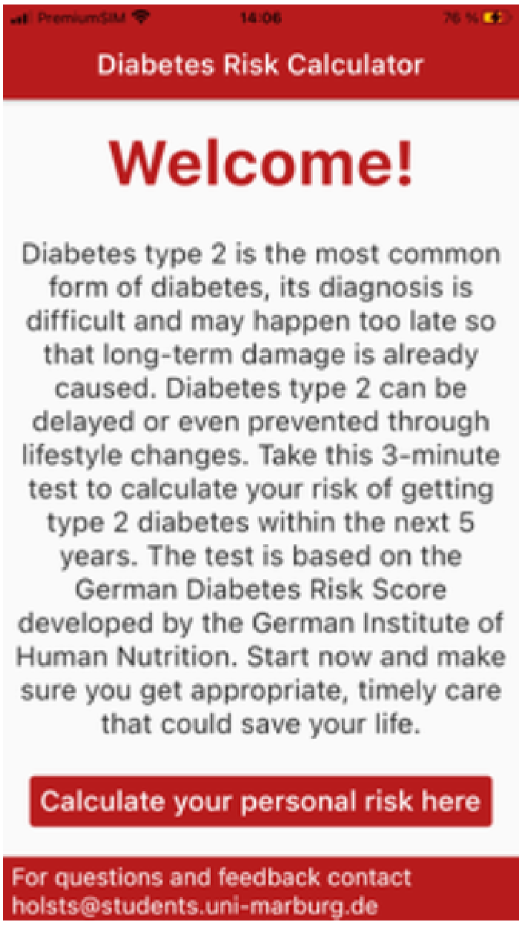
Diabetes Risk Calculator
Welcome!
Diabetes type 2 is the most common form of diabetes, its diagnosis is difficult and may happen too late so that long-term damage is already caused. Diabetes type 2 can be delayed or even prevented through lifestyle changes. Take this 3-minute test to calculate your risk of getting type 2 diabetes within the next 5 years. The test is based on the German Diabetes Risk Score developed by the German Institute of Human Nutrition. Start now and make sure you get appropriate, timely care that could save your life.
Calculate your personal risk here
For questions and feedback contact holsts@students.uni-marburg.de

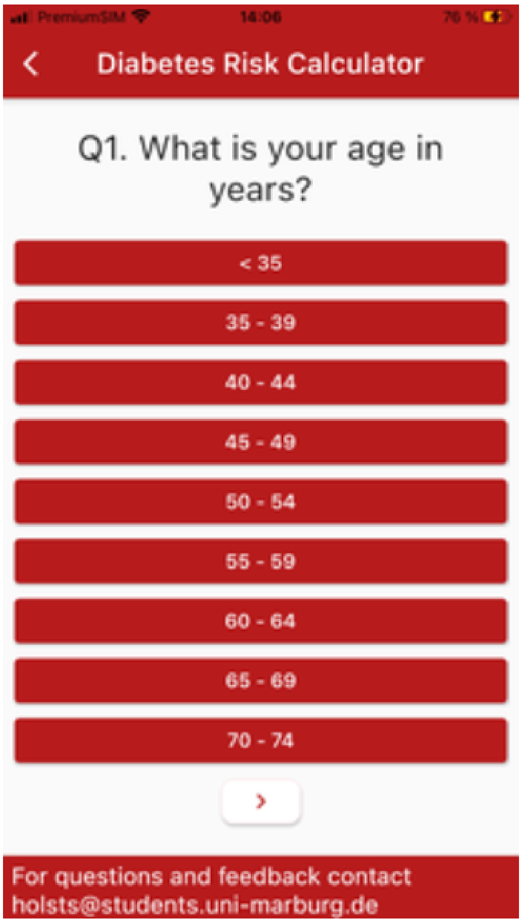
Diabetes Risk Calculator
Q1. What is your age in years?
< 35
35 - 39
40 - 44
45 - 49
50 - 54
55 - 59
60 - 64
65 - 69
70 - 74
For questions and feedback contact holsts@students.uni-marburg.de

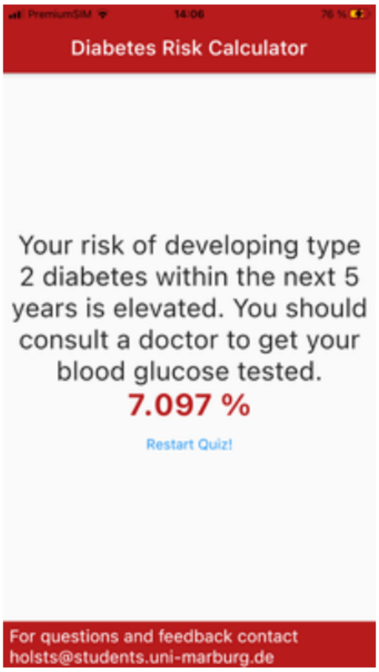

- The build on iOS Simulator and AVD was successful.
- The build on the iOS device was successful.
    - The layout looked beautiful.
    - All tests are passed.
        - The use cases from sprint 2 are repeated.
        - SR9, US11, US12, SR13, SR14, and SR15 (see Requirements Specification - Detailed Specification and Acceptance Criteria) are tested (functionalities like forward and backward navigation).
- 14. June 2021: Android device test:
    - The build was successful.
    - The layout looked beautiful.

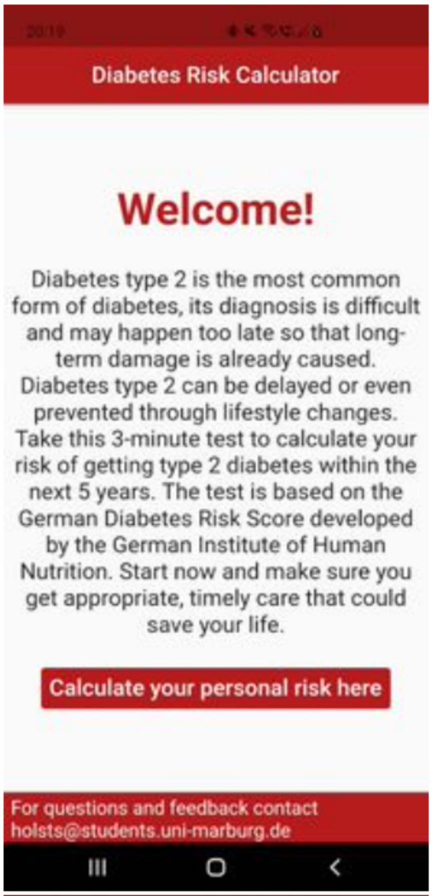
Diabetes Risk Calculator
Welcome!
Diabetes type 2 is the most common form of diabetes, its diagnosis is difficult and may happen too late so that long-term damage is already caused. Diabetes type 2 can be delayed or even prevented through lifestyle changes. Take this 3-minute test to calculate your risk of getting type 2 diabetes within the next 5 years. The test is based on the German Diabetes Risk Score developed by the German Institute of Human Nutrition. Start now and make sure you get appropriate, timely care that could save your life.
Calculate your personal risk here
For questions and feedback contact holsts@students.uni-marburg.de

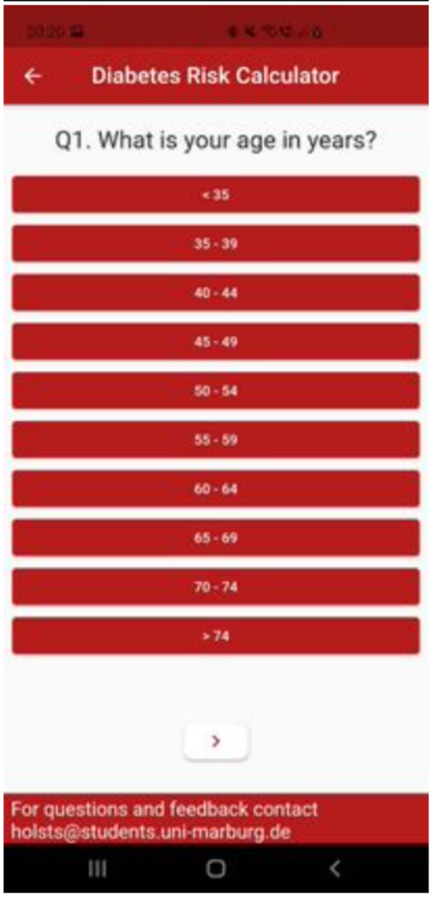
Diabetes Risk Calculator
Q1. What is your age in years?
< 35
35 - 39
40 - 44
45 - 49
50 - 54
55 - 59
60 - 64
65 - 69
70 - 74
> 74
For questions and feedback contact holsts@students.uni-marburg.de

- Succesful build on the Android device (Samsung Galaxy S10+).
  - The url_launcher on the welcome page worked.
  - The url_launcher on the quiz page worked.
  - The url_launcher on the result page worked.
  - Test passed.
- Succesful build on the iOS Simulator (iPad Pro 5th generation).
  - The url_launcher could not be tested on the iOS Simulator since it does not have default email or phone apps installed.
  - Since US6 (see Requirements Specification) defines mobile devices as smartphones or tablets the app has to be tested on a tablet device.
  - The layout is not as beautiful as on the tested smartphones (the font size is quite small) but it is readable. Eventually, the https://ecoup.atlassian.net/browse/DA-26 and the arrangement of the widgets have to be adjusted if the app is supposed to be mainly used on tablets.
  - Test passed.

- The test is passed.

- The change is DoD compliant:

Definition of Done

A requirement is fully implemented sufficing the definition of done if:

- [x] The code is coding guideline compliant.
- [x] The code is consistent, unambiguous, and clearly identifiable.
- [x] A code review was conducted.
- [x] All acceptance criteria are fulfilled.
- [x] A build has been made and deployed on a testing environment.
- [x] All necessary unit, integration, and system tests are passed and documented.
- [x] Traceability to the requirements specification, epics, architecture, tests, and/ or the detailed design document is implemented.
- [x] The implementation does not contradict the architecture description or detailed design document.
- [x] Sprint notes do exist.
- [x] The documentation is complete.

Verification Document Software Release

Software Version	1
Person in Charge	Sabrina Holst
Date	29.06.2021

- The verification is complete and all results are evaluated.
  - The software requirements do not contradict each other, are unambiguous, and clearly identifiable. Sources of the software requirements (to ensure traceability) are:
    - Mühlenbruch K, Joost H, Boeing H, Schulze MB. Risk prediction for type 2 diabetes in the German population with the updated German Diabetes Risk Score (GDRS). Ernaehrungs Umschau. 2014;61(6):90–3.
    - Schulze MB, Hoffmann K, Boeing H, Linseisen J, Rohrmann S, Möhlig M, et al. An Accurate Risk Score Based on Anthropometric, Dietary, and Lifestyle Factors to Predict the Development of Type 2 Diabetes. Diabetes Care. 2007;30(3):510–5.
    - Schulze MB, Holmberg C, Hoffmann K, Boeing H, Joost HG. Kurzfragebogen zur Bestimmung des Diabetesrisikos auf Grundlage des Deutschen Diabetes-Risiko-Scores. Ernaehrungs Umschau Umschau. 2007;54(12):698–703.
    - German Institute of Human Nutrition. Personal test. German Diabetes Risk Score. accessed: 10.05.2021 [Internet]. 2014. Available from: https://www.dzd-ev.de/fileadmin/DZD/PDF/DiabetesRisikotest/141112_DIfE_DRT_Privatperson_2014_en_PRINT.pdf

- These are all used third-party libraries to enable future observation, e.g., for maintenance purpose:
  - `dart:core`
  - `dart:math`
  - `flutter/cupertino.dart`
  - `flutter/flutter_test.dart`
  - `flutter/material.dart`
  - `provider/provider.dart`
  - `url_launcher/url_launcher.dart`
  - The dependencies within the pubspec.yaml:

```
• dependencies:
    flutter:
      sdk: flutter
    cupertino_icons: ^1.0.2
    provider: ^5.0.0
    url_launcher: ^6.0.6
```

- Reliable delivery:
  - The app-release.apk can be provided within Google Drive. Android device users can download it if they allow Google Drive to install unknown apps within the settings. Just search for “Apps” or “Apps&Notifications”, “special app access”, “installation of unknown apps”, and trust the source Google Drive.
  - To install the app on iOS devices you need to connect the device to a Mac and install the app through Xcode.
- Rest anomalies:
  - The layout could be improved for tablet devices.
  - The sensitivity and specificity within the non-functional requirements have to be tested through a clinical study (but the sensitivity and specificity of the GDRS suffice them).
- From the development point of view, the software is complete.

- Ran all automated tests within the project's test directory.
  - All tests are passed.
- The acceptance criteria of US5 and US6 (High-level requirement 6) are fulfilled.
- No open To-dos.
- The requirements are DoD compliant:

Definition of Done

A requirement is fully implemented sufficing the definition of done if:

- [x] The code is coding guideline compliant.
- [x] The code is consistent, unambiguous, and clearly identifiable.
- [x] A code review was conducted.
- [x] All acceptance criteria are fulfilled.
- [x] A build has been made and deployed on a testing environment.
- [x] All necessary unit, integration, and system tests are passed and documented.
- [x] Traceability to the requirements specification, epics, architecture, tests, and/ or the detailed design document is implemented.
- [x] The implementation does not contradict the architecture description or detailed design document.
- [x] Sprint notes do exist.
- [x] The documentation is complete.

Verification Document Change Request 1

Software Version	1
Person in Charge	Sabrina Holst
Date	17.06.2021

- **Relevant Software Configuration and Tools:**
  Android Studio, Flutter, Dart, XCode, AVD, iOS Simulator (iPhone 12 pro Max) (for more details see https://ecoup.atlassian.net/wiki/spaces/DA/pages/370049134/Verification+Documents#Android-Studio-Setup)

  Test devices: Samsung Galaxy S10+ (Android version 11)
- Verification of https://ecoup.atlassian.net/wiki/spaces/DA/pages/386072678/Change+Requests#Change-Request-1
- The build on the iOS Simulator and AVD were successful and only two decimal places are shown.

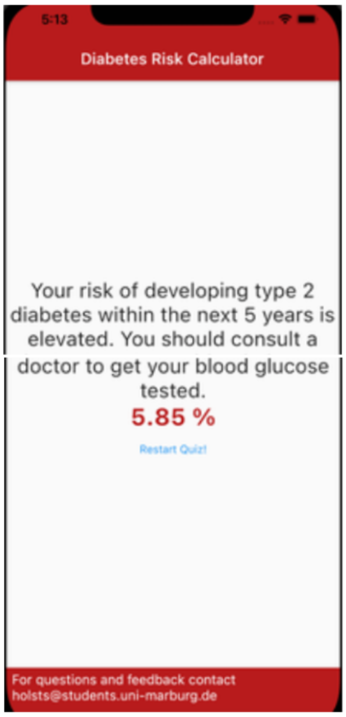

- Ran all automated tests.
  - All automated tests are passed.
- Installed the app-release.apk on the Android test device.
  - The test is passed.

B.8 Change Requests

- Change Request 1

Change Request 1

Person in Charge	Sabrina Holst
Date	17.06.2021
Problem Report	None - the change request is not related to a problem.
Status	APPROVED

- The result on the result screen currently has three decimal places.
- Two decimals would improve the readability and the accuracy of the decimal places does not influence the result.
- Change request: Adjust the result within the result service to two instead of three decimal places.
- The change request was approved by A.C. Hauschild (17.06.2021).
- Cange request realized on the 17.06.2021, see https://ecoup.atlassian.net/wiki/spaces/DA/pages/370049134/Verification+Documents#Verification-Document-Change-Request-1 for its verification.
